# Physiological determinants of cortical P100 responses in pattern visual evoked potentials: a scoping review

**DOI:** 10.3389/fnins.2026.1821657

**Published:** 2026-05-26

**Authors:** Łukasz Lisowski, Jolanta Lisowska, Łukasz Łabieniec, Mateusz Zarzecki, Dominik Zalewski, Iwona Obuchowska, Joanna Konopińska

**Affiliations:** 1 Department of Ophthalmology, Medical University of Bialystok, Bialystok, Poland; 2 Individual Medical Practice, Bialystok, Poland; 3 Faculty of Physics, University of Bialystok, Bialystok, Poland; 4 Lens Center for Eye Diagnostics and Microsurgery, Olsztyn, Poland

**Keywords:** aging brain, cortical processing, ISCEV standards, P100 amplitude, P100 latency, pattern visual evoked potential, retinal image quality, visual electrophysiology

## Abstract

**Background:**

Pattern visual evoked potentials (pattern VEP) are widely used for functional assessment of the visual pathways. The P100 component represents the principal clinical parameter owing to its relative interindividual stability and diagnostic value. However, both latency and amplitude are modulated by multiple physiological and environmental factors, which complicates interpretation and the establishment of reliable reference standards. This scoping review aimed to systematically map determinants of P100 parameters in healthy individuals.

**Main text:**

The review was conducted in accordance with PRISMA-ScR and Joanna Briggs Institute methodology. Databases were searched for studies published between 2015 and 2025 that examined biological, refractive, anthropometric, metabolic, or environmental influences on pattern VEP parameters in healthy populations. Owing to methodological heterogeneity, findings were synthesized descriptively. Thirty-nine studies met the inclusion criteria. Age emerged as the most consistent determinant of P100 parameters. Latency followed a non-linear trajectory across the lifespan, with shortening during maturation, stabilization in early adulthood, and progressive prolongation after approximately 40 years of age, whereas amplitude generally declined with aging. Sex differences predominantly affected amplitude, with women typically demonstrating higher P100 or N75–P100 amplitudes in adult populations; latency differences were less consistent and often minimal in paediatric cohorts. Retinal image quality exerted a strong dose-dependent effect on P100 parameters: increasing refractive blur and higher-order aberrations were associated with progressive latency prolongation and amplitude reduction, particularly for small check sizes. Ocular dominance showed no clinically meaningful interocular asymmetry. Metabolic disturbances were associated with prolonged latency in selected populations, whereas anthropometric variables such as head size and height demonstrated weak or inconsistent associations. Among environmental factors, acute alcohol intake prolonged P100 latency, while moderate caffeine consumption had no significant effect.

**Conclusion:**

Age and retinal image quality represent the primary physiological determinants of P100 latency and amplitude in healthy individuals. Most other modifiers exert modest or context-dependent effects. Consideration of these variables is essential for accurate interpretation of pattern VEP recordings and for establishing reliable local reference standards consistent with ISCEV recommendations.

## Background

Visual evoked potentials (VEP) test is a non-invasive method designed to record bioelectrical activity of the visual cortex, which is generated in response to a light stimulus. This technique allows for functional assessment of the visual pathway—from the axons of retinal ganglion cells to the cortical visual centres in the occipital lobe.

The VEP response depends on both optical and neural components. When a light stimulus passes through the optical media of the eye, it stimulates photoreceptors (cone cells and rod cells), and then the signal is transmitted by interneurons to ganglion cells. The axons of ganglion cells form the optic nerve, which runs to the optic chiasm, and further to the lateral geniculate body; from there, the optic radiation transmits information to the visual cortex (Moraes, 2013). The primary visual cortex (V1; Brodmann area 17) is located near the calcarine sulcus and plays a central role in early cortical visual processing. However, visual information processing is not strictly serial. Visual signals are transmitted through parallel pathways, including the magnocellular and parvocellular systems, which differ in their functional specialization and contribute differentially to the processing of motion, temporal resolution, form, and fine spatial detail. After initial cortical input, information is further processed through secondary and tertiary visual areas, which participate in higher-order analysis and integration with information from other sensory modalities (Creel, 2012).

The VEP test result is presented in the form of a curve consisting of positive (P100) and negative (N75 and N135) deflections. Basic interpretation of such recording includes the assessment of peak time (which in this study is used interchangeably with the traditionally used term “latency”) and the amplitude of main components of the response (Seok et al., 2018). Another significant element of this interpretation is the assessment of symmetry of the interocular recordings, as well as the comparison of responses recorded over the right hemisphere and left hemisphere. In the case of normal conditions, it is expected to obtain high symmetry of responses in both above-mentioned approaches, while the peak time and amplitude values should be within the range of accepted reference norms.

Determination of reference values constitutes an essential stage in the scope of preparation of electrophysiology laboratory for clinical practice. VEP parameters are susceptible to the influence of many different factors, including technical factors (equipment, monitor type, stimulation parameters, and recording conditions), environmental factors (e.g., background lighting), as well as factors associated with the examined person (among others: age, sex, anthropometric features, refractory state, visual acuity, pupil size) (Thompson et al., 2023). As a result, the International Society for Clinical Electrophysiology of Vision (ISCEV) recommends the preparation of local normative values, which should be adapted as best as possible to the applied equipment, recording protocol and tested population, while maintaining compliance with applicable standards (Šuštar Habjan et al., 2025).

The literature includes numerous publications that present the standards developed in various centres and regions of the world (including Indonesia, Iran, India, Sri Lanka, Bulgaria, Poland) (Ekayanti et al., 2021; Mahjoob et al., 2019; Bhimte, 2017; Dahanayake et al., 2020; Lisowski et al., 2026). These studies—which include studies conducted in accordance with the current ISCEV standards as well as earlier normative studies—emphasise the significance of local validation of the VEP parameters, and they indicate that there are biological as well as demographic determinants for variability of the responses.

This review was not intended as a historical overview of all major studies on pattern VEP physiology, but was conceived as a synthesis of contemporary evidence from the last decade, interpreted in the context of selected seminal publications relevant to the field. This temporal restriction was adopted to improve consistency with current ISCEV-based methodological frameworks and contemporary standards of clinical interpretation.

With this in mind, it seems reasonable to systematise the knowledge concerning individual factors that—under physiological conditions—may modify the VEP recordings. The aim of this study is to present the influence of selected variables associated with the examined person (among others: age, sex, anthropometric and refractive characteristics) on the parameters of VEP responses. Such a comparison may constitute a practical supplementation to the electrophysiology laboratories because it can facilitate the critical interpretation of results and distinguish physiological changes from potentially pathological changes.

## Methods

### Research project

This study was elaborated in the form of a scoping review with an aim of systematizing the available scientific data on biological and environmental factors which impact visual evoked potentials (VEP) in healthy individuals. The review was performed in line with the PRISMA Extension for Scoping Reviews (PRISMA-ScR) (Tricco et al., 2018) guidelines and based on the scoping review methodology recommended by the Joanna Briggs Institute (JBI). The study protocol was deposited and registered on the Open Science Framework (OSF) and has since then been publicly available at: https://osf.io/kcvbx (DOI: 10.17605/OSF.IO/KCVBX) (Lisowski, 2026).

The use of a scoping review format was justified by the substantial heterogeneity of the available studies, encompassing differences in study designs, stimulation and recording parameters, as well as the reported VEP response measures, which prevented the conduct of a quantitative synthesis.

### Literature search strategy

The below databases were browsed in pursuit of comprehensive literature: PubMed (MEDLINE), Web of Science Core Collection, Science Direct, The Cochrane Library, and Google Scholar. The search period range covered publications from January 1, 2015, to December 2025, allowing for a focus on studies published after the introduction of the ISCEV standard for pattern-evoked potentials (Odom et al., 2016) and ensuring greater methodological homogeneity of the examined material.

The search was narrowed down solely to publications in English. Depending on the database, combinations of controlled terms (for instance, Medical Subject Headings—MeSH) and free-text terms were applied, encompassing the concepts linked to VEP (among others, visual evoked potentials, VEP, PVEP, pattern VEP) and the biological and environmental factors analyzed, such as age, sex, ocular dominance, nutritional status, alcohol and caffeine consumption.

Results were sorted by relevance by means of the Google Scholar and subsequently, the first 200 records were analyzed based on titles and, where appropriate, abstracts. The browse search through Cochrane Library database was conducted directly in the search interface albeit, due to a lack of eligible records no data were ultimately exported to the reference manager. Furthermore, a “snowball” method was availed of, i.e., analysing reference lists of publications eligible for further review; this procedure did not however result in an identification of additional studies fulfilling the inclusion criteria.

Even though the primary comparative analysis covered publications published in the years 2015–2025, the selected earlier studies were considered in the main text solely for the purpose of presenting the historical research basis and primary observations regarding the physiological variability of VEP responses. These publications were contextual in character and thus were included in neither the comparative analysis nor the tabulated synthesis of the results; reference was drawn to them exclusively for the purpose of better comprehension of the biological mechanisms and evolving methodological approaches in pattern VEP studies.

Publications representing guidelines, standards, and consensus statements were used solely as methodological references (background); they were not considered in the empirical data synthesis or the tabulated summary of the results.

### Criteria of inclusion and exclusion

Studies meeting the following criteria were included in the review:

- original research involving healthy individuals or healthy participants of a control group,
- the use of visual evoked potentials as a method for functional assessment of the visual pathway,
- analysis of at least one of the predefined biological or environmental factors,
- publication in English,
- full text availability.

The following were excluded from the review:

- non-human studies,
- reviews, guidelines, methodological consensuses, and standards,
- case reports and case series with limited comparative value,
- duplicate publications or publications reporting the same clinical data.

### Study selection process

All identified records were imported into a literature management program, followed by the removal of duplicates. Post conduct of deduplication, 1,762 records were analyzed at the title and abstract assessment stage. On this basis, 110 publications were eligible for full-text review; all of the full texts were retrieved and subjected to a detailed analysis.

**Figure 1:**
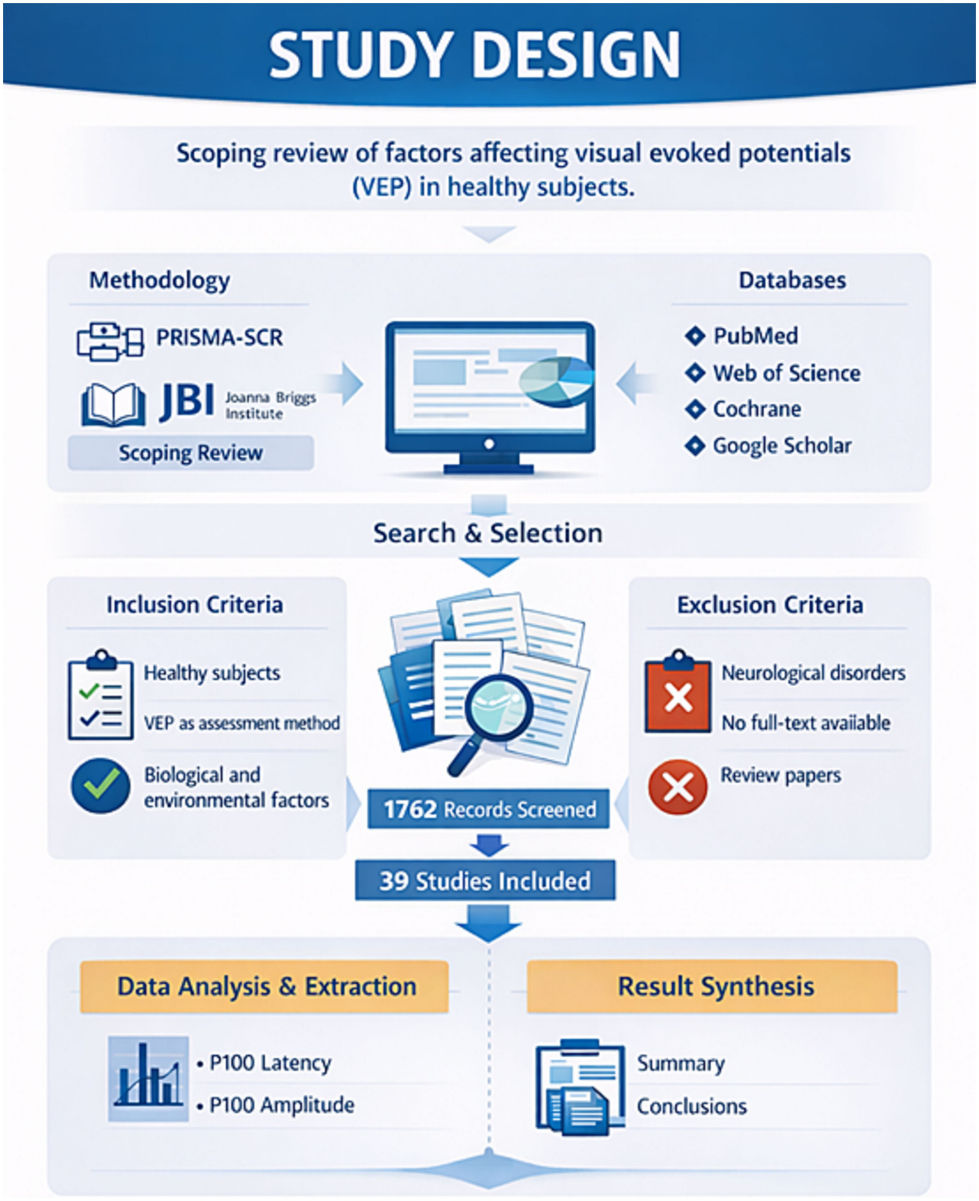
Scoping review project flowchart of factors influencing visual evoked potentials (VEP) in healthy individuals, including the search strategy, study selection, and analysis of P100 wave parameters.

Once the full-text review had been completed, 39 studies that met all eligibility criteria were included in the substantive analysis. The remaining publications were rejected due to, among other reasons, inappropriate study type (e.g., guidelines, standards), lack of a healthy individuals’ population, insufficient description of methodology, or lack of relevant VEP response parameters. The study selection process has been presented in the form of a flow diagram compliant with the PRISMA-ScR guidelines (Figure 1).

### Data extraction and synthesis

Data extraction was performed using a standardized form, including characteristics of the studied population, stimulation and VEP recording parameters as well as the reported results, with particular emphasis placed on P100 wave latency and amplitude. A detailed methodological overview of the included studies is provided in Supplementary Table S1, including population characteristics, stimulus parameters such as checkerboard size, contrast, luminance, stimulation field and reversal rate, as well as recording parameters including electrode montage, filtering, sweep duration and the number of averaged responses, where these data were reported by the original authors. This table was intended to facilitate comparison of the methodological background underlying the reported P100 latency and amplitude findings. The collected data were subsequently subjected to descriptive synthesis and presentation in tabular and narrative formats. Meta-analysis was neither planned nor conducted for this study.

### Normative P100 values in healthy adult populations

To facilitate direct comparison of P100 parameters across studies, a summary of normative values reported in healthy adult populations is presented in Table 1. Only studies using pattern-reversal stimulation and reporting quantitative values of P100 latency and/or amplitude were included.

**Table 1:** Statistical characteristics of P100 in normative pattern-reversal VEP studies in healthy adults.

Author (Year)	Title	Population	Eye/analysis unit	Check size	Luminance/contrast	P100 latency (ms)	P100 amplitude (μV)	Methodological remarks
Mermeklieva (2019)	*Reference values of pattern reversal visual evoked potentials in Bulgarian population*	Healthy adults, 27–51 yrs.; *n* = 47	Right eye used for final analysis	0.25°/15′	High-contrast black–white; luminance n/r	107.4 ± 4.8	10.73 ± 5.12	Oz–Fz; 15° field
Mermeklieva (2019)	*Reference values of pattern reversal visual evoked potentials in Bulgarian population*	Healthy adults, 27–51 yrs.; *n* = 47	Right eye used for final analysis	1°/60′	High-contrast black–white; luminance n/r	102.0 ± 4.6	10.22 ± 4.94	Oz–Fz; 30° field
Dahanayake et al., 2020	*Normal values of pattern reversal visual evoked potentials (PRVEP) and pattern electroretinography (PERG) in healthy adults in Sri Lanka*	Healthy adults, 20–62 yrs.; *n* = 50	RE and LE reported separately	1° and 0.25°	50 cd/m^2^; ≥80% contrast	RE: 102.38 ± 5.86; LE: 101.61 ± 6.30	RE: 5.01 ± 3.39; LE: 4.29 ± 3.32	Values not separated by check size
Ekayanti et al., 2021	*Normative values of visual evoked potential in adults*	Healthy adults, 18–65 yrs.; *n* = 120	RE and LE reported separately	26′	80% contrast; room lighting 60 lx	RE: 104.1 ± 3.4; LE: 104.6 ± 3.4	RE: 10.3 ± 5.4; LE: 9.8 ± 4.7	Normative adult cohort
Sawaya et al. (2017)	*Pattern reversal visual evoked potentials in adults: variability with age*	Healthy adults, 20–92 yrs.; *n* = 81	Both eyes included; age-stratified values	1° / 60′	50 cd/m^2^; high contrast >50%	103.3 ± 4.5 to 118.1 ± 7.79	5.5 ± 2.41 to 11.3 ± 5.79	Values vary by age decade
Baumgarten et al. (2022)	*Fullfield and extrafoveal visual evoked potentials in healthy eyes: reference data for a curved OLED display*	Healthy adults; phakic eyes, *n* = 138	One eye per subject	1.4°	Black 0.58 cd/m^2^; white 105.8 cd/m^2^; 99% contrast	102.58 ± 7.26	15.66 ± 6.42	FF-PR-VEP; curved OLED
Baumgarten et al. (2022)	*Fullfield and extrafoveal visual evoked potentials in healthy eyes: reference data for a curved OLED display*	Healthy adults; phakic eyes, *n* = 138	One eye per subject	20.4′	Black 0.58 cd/m^2^; white 105.8 cd/m^2^; 99% contrast	103.81 ± 7.77	16.30 ± 7.53	FF-PR-VEP; curved OLED

Across studies conducted under conditions broadly consistent with ISCEV recommendations, mean P100 latency typically ranged from approximately 100 to 105 ms. In contrast, amplitude values demonstrated substantially greater variability, generally ranging from approximately 5 to 16 μV.

This variability is likely attributable to differences in stimulation parameters, including check size, luminance, and contrast, as well as population characteristics and recording conditions. These findings highlight the importance of careful methodological standardization and support the need for locally established normative reference values.

#### Age

The development and aging of the visual system find their reflection in the characteristics of pattern visual evoked potentials (pattern VEP) responses, particularly in terms of the latency and amplitude of the principal components (N75, P100, N135). Literature data encompassing both normative studies, conducted in line with current standards and earlier developmental studies, indicate clear age-related differences spanning the lifespan—from the neonatal period and infancy, through childhood and adolescence, to adulthood and later life phases. In this section, in addition to studies which meet the inclusion criteria for the comparative analysis (2015–2025), selected earlier works have also been quoted, which are crucial when describing the physiology of the development and aging of the visual system; these publications provide a context function.

##### Neonatal and infancy period

The neonatal and infancy period is featured by rapid maturation of the visual pathways and an intensive myelination. Thompson et al. (2023) in a study covering 649 healthy participants aged from 2 weeks to 16 years, demonstrated that age is the main factor modulating VEP pattern parameters in the first months of life. P100 latency (referred to by the authors as *peak time*) was significantly shortened, stabilizing around 27 weeks of age for large checkerboard squares (50′ and 60′) and around 34 weeks of age for small squares (12′, 12.5′, and 15′). These values corresponded to the typical adult ranges (87–115 ms and 96–131 ms, respectively). Whilst the P100 amplitude exhibited significant interindividual variability and did not demonstrate a clear, systematic age-dependent trend.

Comparative analysis of data from three centres (Great Ormond Street Hospital in London, University of Pécs Medical School, and the Royal Hospital for Children in Glasgow) constituted a significant element of the study. The authors demonstrated that differences in technical recording parameters, such as the pattern reversal frequency (1.1–3.75 Hz) or the acquisition trigger (e.g., from the corner of the CRT screen instead of the centre of the refresh rate), led solely to minor shifts in latency, albeit not altering the overall developmental pattern of the VEP response. Even though the age distribution of participants varied from centre to centre, and the observed differences were not reproducible for both checkerboard square sizes and thus, were relatively small in range, the authors considered the data sufficiently consistent to combine them into a single common reference set. These results comply with the high comparability of VEP pattern responses while maintaining ISCEV technical standards and underscore the importance of precise reporting of methodological parameters in electrophysiological studies.

Similar observations were reported by Torres-Espínola et al. (2018) who demonstrated that between 3 and 18 months of age, healthy infants experienced a shortening of P100 latency with relative stability of response amplitude for large checkerboard squares, whereas an increase in P100 amplitude was observed for the smallest patterns (7.5′). These results correspond to the findings of Nieto-Ruiz et al. (2019) who observed substantial shortening of the P100 latency between 3 and 12 months of age, particularly for small checkerboard squares (15′ and 7.5′), with a simultaneous concomitant decrease in amplitude, more pronounced in case of larger stimulation fields (120′, 60′, and 30′). Jensen et al. (2019) reported higher amplitude values of the P1 component (corresponding to P100 in the pattern VEP nomenclature) in 6-month-old infants compared to 36-month-olds. The authors suggested that the observed decrease in amplitude during development may reflect not only the functional maturation of the visual system but also anatomical changes, such as skull bone thickening and the relocation of cortical sources.

##### Childhood

Once the intensive maturational phase is over, the visual system enters a period of relative stability spanning childhood and adolescence. Thompson et al. (2023) showed that from the moment of latency stabilization in the first year of life until late adolescence (age of 16), P100 latency does not undergo any substantial changes; meanwhile amplitude shows only individual variability with a slight downward trend. This result indicates that childhood and adolescence are featured by relative electrophysiological stability of the visual system. Jiang et al. (2023) confirmed that the stability of amplitudes in a group of children aged 4–13 years, albeit they noted a slight, gradual increase in the latencies of the N75, P100, and N135 components with age. Patterson Gentile et al. (2021) described a gradual decrease in P100 amplitude in adolescents aged 11–19 years and an increase in N135 amplitude at stable latencies. Kovarski et al. (2016) when comparing adolescents (11–16 years old) and adults (20–48 years old), revealed lower P100 and N75 amplitudes in adults and significantly shorter N75 latency in adults (53.9 ± 4.6 ms) in comparison to adolescents (57.9 ± 3.7 ms), with no significant differences in P100 latency.

##### Adulthood

During young adulthood, spanning the ages from 20 to approximately 40, a final phase of latency shortening is observed, followed by a phase of greatest stability in VEP pattern parameters (Benedek et al., 2016). Benedek et al., having conducted an analysis of a population of individuals aged 5 to 84 years, demonstrated that P100 latency shortened to approx. The 30s and then gradually lengthened with age. A similar pattern was observed for the N135 component, whose latency shifted in parallel with P100, while N70 latency increased quasi-monotonic throughout the entire age range in question. Changes in P100 and N135 latencies were long-lasting, reaching their minimum values only around the 30s and indicating a prolonged process of functional maturation of the visual pathways. The P100 amplitude, on the other hand, revealed a nearly monotonic decline from childhood to old age, without a period of explicit stabilization. Moreover, the authors noted a higher incidence of double-peak P100 components in older age groups, interpreting this as a physiological effect of aging. The analysis conducted for different stimulation conditions (different contrasts, luminance, and stimulus colours) presented no significant differences in the course of developmental changes, and the age-dependent VEP responses were similar across all conditions. These results indicate that after reaching the minimum latency in the third decade of life, gradual aging-related trends may become apparent, which in some studies become more explicit in the subsequent decades of life.

The observations regarding young adulthood are complemented by the results of study Mermeklieva (2019), whereby the effect of age on VEP pattern parameters in a group of 47 healthy adults aged 27–51 was evaluated. The authors demonstrated that VEP parameters remain highly stable within this particular age range—no material changes were detected in the latency or amplitude of the principal components (N75, P100). Solely a minor trend toward shorter latencies and lower amplitudes was observed in younger individuals, however with no statistical or clinical significance. This effect was observed only in case of a small number of recording electrode positions which proves that age-related differences were local in nature rather than general throughout the recordings. The N75–P100 amplitude and P100 latency were confirmed to be the most stable parameters with the least noted variability in the adult population. It is worth emphasizing that the observed direction of changes (lower amplitude with shorter latency in younger subjects) deviates from the typical results of most population-based studies, whereby the P100 amplitude tends to decrease whilst latency increases with age. The limited age range of the participants (27–51 years) may stand behind the absence of the classic effect of visual aging in this study.

After the age of 40, the first signs of visual aging begin to become apparent. Ekayanti et al. (2021) indicated that in the adult population aged 18–65 years, P100 latency remains relatively stable; meanwhile amplitude decreases in the oldest age group (61–65 years). Lek et al. (2019) showed that the key age effect is an increase in P100 latency by approximately 10 ms on average in older adults, regardless of the contrast, whereas amplitude remains stable and, at higher contrasts, may be even greater than in younger adults, indicating a complex relationship between age, stimulus contrast, and response size. The authors suggested that the increased amplitude of the VEP pattern in older adults may stem from a change in the balance between excitatory and inhibitory processes in the visual cortex (imbalance of excitatory/inhibitory processes), rather than from a simple reduction in cortical inhibition. An impaired contrast response function may therefore reflect complex changes in the mechanisms of regulation of neural activity, leading to a relative increase in cortical responses to high-contrast stimuli.

##### Old age

In the population of people over 60 years of age, shifts in VEP waveform parameters become the most transparent. Baumgarten et al. (2022) described a systematic increase in P100 latency and a decrease in N75–P100 amplitude by an average of approx. 0.4% per annum. The analysis included individuals aged 10 years and older (groups: 10–19, 20–39, 40–59, and ≥60 years), whilst the average age of the oldest group was approximately 70 years, which enabled assessment of the full course of maturation and aging of the visual pathways. Latency in the youngest group (10–19 years) was higher than in the group of adults aged 20–39, where the shortest P100 values were noted. In the later decades of life, a systematic increase in latency, estimated at approximately 0.07 ms per year, was observed. The N75 and P100 components showed a clear age dependence—the N75 latency shifted linearly, while the P100 took a *U*-shaped course, reflecting the shortening of latency during adolescence, stabilization in young adulthood, followed by re-lengthening in older age. The effect of age was also shown to be dependent on stimulation parameters. In the case of large checkerboard elements (1.4°), N75 latency did not show any significant age-related differences, whilst in case of smaller elements (20.4′), an age-proportional increase in N75, P100, and N135 latencies was reported. Moreover, the N75–P100 amplitude was modulated by age—for large pattern elements, decreasing by an average of 0.4% per annum, whereas for smaller elements, the differences did not reach a statistically-significant level. The authors indicated that the obtained results confirm the age dependence of both main components of the VEP pattern (N75 and P100), with the nature of the changes varying depending on the life stage and type of stimulation. Early visual development is featured by the shortening of latencies and a greater amplitude variability, while aging is characterized by a gradual increase in latency and a decrease in amplitude, particularly in responses generated by large checkerboard squares. This finding may reflect stimulus-dependent differences in the functional visual pathways engaged by different pattern conditions; however, the available evidence does not allow a definitive mechanistic explanation.

Sawaya et al. (2017) confirmed the existence of a linear relationship between age and N75, P100 as well as N145 latencies in a population of individuals aged 20–92 years, indicating that latencies were longer in older age groups. It was observed that latencies shortened in the fifth decade of life, after which they lengthened again in the higher age groups. Additionally, the amplitude of the N75–P100 complex was found to decrease with age.

The relationship between age and amplitude of the pattern VEP response is also reflected in the structure of the visual cortex. When Slapø et al. (2023) analysing the relationship between pattern VEP wave parameters and the structure of the visual cortex (V1) in a group of 307 healthy individuals aged 18–85 years, demonstrated a significant decrease in P100 amplitude with age. They noted a significant positive correlation between P100 amplitude and V1 area, however, found no correlation between P100 and the thickness of this structure. These results indicate that the reduction in P100 amplitude in older individuals may reflect a reduced neuronal activity resulting from aging in the visual cortex. Hence, structural changes within V1 represent a significant factor modulating the functional parameters of the VEP pattern response in healthy individuals. The authors explain the relationship between P100 amplitude, age and the surface area of the V1 visual cortex from the neurophysiological and morphological perspective. As the brain ages, the surface area of V1 decreases, which is associated with fewer cortical columns. This, in turn, leads to weaker summation of post-synaptic potentials (PSP) generated by V1 pyramidal neurons. Since the P100 amplitude in the VEP pattern reflects the combined activity of these neurons, a smaller number of synchronized unit’s results in a reduced amplitude. Furthermore, the level of axonal myelination in V1 may correlate with its surface area—improved myelination allows for more synchronous impulse conduction, which increases potential summation and leads to a larger P100 amplitude. As aging progresses, along with the cortical surface area decreases and the myelination decreases, neuronal synchronization deteriorates, explaining the drop in P100 amplitude in older individuals. In conclusion, the relationship between age, P100 amplitude, and V1 surface area stems from the fact that aging leads to a reduction in the number of columns and the degree of myelination in the visual cortex, which limits the summation of postsynaptic potentials and, consequently, weakens the VEP response.

The cumulatively presented data indicate that age is one of the strongest factors modulating VEP pattern parameters, whilst the nature of these changes is dependent on the life stage and type of stimulation. Older developmental studies provide a biological context for the results of studies conducted in accordance with current ISCEV standards and enable their correct interpretation.

#### Sex

Sex is one of the most frequently analyzed biological factors that may influence pattern VEP response parameters, although research results are not entirely consistent in this respect. Some studies indicate significant differences between women and men, while others do not confirm them, and the extent of the observed effects may depend on both the age of the participants and the applied technical recording conditions. The analysis primarily included studies that met the inclusion criteria for the tabular synthesis, while selected earlier studies were cited as the context for the most frequently discussed hypotheses explaining sex-related differences in pattern VEP parameters.

In the study by Sharma R. et al., Sharma et al. (2015) the impact of sex on pattern VEP parameters was assessed in 100 healthy young adults (50 women and 50 men) aged 17–20. Responses in women were characterized by shorter latencies and higher P100 wave amplitudes compared to men. Sex differences were also statistically significant in the scope of latencies of the N70 and N155 waves. The average P100 latency amounted to 88.3 ± 8.8 ms (left eye) and 88.8 ± 9 ms (right eye) in women, and 93.2 ± 10.7 ms (left eye) and 93.4 ± 10.6 ms (right eye) in men, respectively (*p* < 0.05). The P100 amplitude was significantly higher in women – 6.4 ± 0.7 μV (left eye) and 6.4 ± 0.7 μV (right eye)—compared to men, who had 5.7 ± 0.5 μV and 5.7 ± 0.5 μV (*p* < 0.01). The authors noted that the study included a narrow age group of young adults, which limits the generalizability of the results to populations in other age ranges. The authors interpreted the observed sex differences as potentially related to anatomical factors (smaller head size and shorter conduction path in women) and endocrine factors, drawing reference to previous reports of shorter P100 latencies in pregnant women.

In a study conducted by Benedek et al. (2016) the relationship between sex and VEP waveform parameters was analyzed in a population of 115 healthy individuals aged 5–84 years (87 females and 28 males). The authors recorded the N70, P100, and N135 components using various stimulation conditions (97% contrast, 6% contrast, and red-green checkerboard stimulation). The analysis proved that sex was a significant factor determining all VEP components (N70, P100, N135), regardless of the stimulus type (*p* < 0.001). The authors noted that women were characterized by shorter latencies and larger response amplitudes compared to men, confirming previous reports of faster conduction and greater excitability of the visual cortex in women. The interactions between sex, age, stimulus contrast, and pattern size were not statistically significant (*p* > 0.05), indicating that the impact of sex on VEP parameters is not modified by age or stimulation conditions. In spite of the study population having been uneven in terms of sex (approximately 76% female), the results indicate that sex differences in VEP patterns were observed across the entire age range and stimulation type. This signifies that women tend to generate stronger and faster visual responses, which may be due to anatomical and neurophysiological factors.

In the study by Baumgarten et al. (2022) the influence of age, sex and lens status on VEP pattern parameters was tested using a modern, curved OLED (Organic Light-Emitting Diode) display as the stimulator. Both full-field (FF-PR-VEP) and extrafoveal (EF-P-ON/OFF-VEP) responses were analyzed in 162 healthy individuals (69 males and 93 females) above the age of 10 years. The authors demonstrated that sex significantly affects VEP parameters, regardless of age. For the FF-PR-VEP (ss 1.4°), the P100 latency was on average 2.84 ms shorter in women than in age-matched men, suggesting that age did not exacerbate the sex difference. Furthermore, the N75–P100 amplitude was 33.9% larger in women when stimulated with a larger checkerboard size (ss 1.4°). When stimulated by means of smaller checkerboard sizes (ss 20.4′), the differences were even more pronounced – the N75–P100 amplitude was 53.7% larger in women (*p* < 0.001). In the EF-P-ON/OFF-VEP study, women also showed larger C1–C2 amplitudes for both checkerboard sizes used (1.4° and 2.8°; *p* < 0.001).

Most classical VEP pattern analyses focus on assessing the basic signal parameters, such as P100 latency and amplitude, demonstrating that in many studies, these values are shorter and larger in women than in men, respectively. Nevertheless, in the study by Patterson Gentile et al. (2021) involving 155 healthy participants aged 11 to 19 years, an alternative analytical approach was applied—principal component analysis (PCA)—which allowed for the assessment of the entire temporal shape of the visual response, not just its selected point features. It demonstrated that, in addition to the differences in amplitude, pattern VEP waves also differed morphologically between the sexes: a narrower, steeper P100 peak was observed in girls and young women, while a broader, lower-amplitude peak was observed in boys and young men. These results form an expansion of traditional observations, indicating that sex-related differences in pattern VEP encompass not only the quantitative aspects (latency, amplitude) but also the qualitative differences in the shape of the cortical response, which may reflect different mechanisms of maturation and functional organization of the visual system in girls and boys during the period of adolescence.

In the study by Slapø et al. (2023) the relationship between pattern VEP response parameters and the structure of the primary visual cortex (V1) was analyzed in healthy individuals and patients with affective and schizophrenic disorders, including 307 healthy adults (59% female, age 18–85 years). The analysis showed that sex had a substantial effect on P100 amplitude—men had lower amplitudes of this component compared to women. No effect of sex was observed on the thickness or surface area of the primary visual cortex (V1). These results are consistent with previous literature reports indicating that women have larger amplitudes and shorter P100 latencies, which may be due to anatomical or hormonal differences influencing visual cortex excitability. In conclusion, the study by Slapø et al. (2023) demonstrated a separation between the functional differentiation of the pattern VEP response and comparable structural parameters of the primary visual cortex (V1) in women and men.

The study by Dotto et al. (2017) assessed the effect of sex on pattern VEP parameters in 54 healthy adults aged 18–74 years. The analysis of full-field pattern-reversal VEP responses showed that women had significantly larger N75–P100 amplitudes compared to men, particularly for smaller checkerboard squares (15′)—an average of 12.8 μV in women vs. 8.6 μV in men. For larger squares (60′), the amplitude differences were statistically significant, although slightly smaller. P100 peak times were slightly shorter in women than in men (approximately 2–3 ms difference), both in monocular and binocular conditions, but only for stimulation with smaller checkerboard squares (15′). N135–N75 interval values were similar between the sexes, though a trend toward shorter response duration was recorded in women. No significant differences were detected in interocular amplitude difference (IAD) or binocular summation ratio (BSR) between men and women.

In the study, Mermeklieva (2019) assessed the effect of sex on pattern VEP waveform parameters in a group of 47 healthy adults (21 men, 26 women) aged 27–51 years. The author demonstrated that the amplitude of the N75–P100 component was greater in women, whilst the P100 latency was shorter compared to men. These differences reached statistical significance (*p* < 0.01) only at some recording electrode positions, indicating that the effect of sex was not uniform across pattern VEP recordings.

In a study by Mahjoob et al. (2019) the impact of physiological factors, including sex, on pattern VEP parameters was evaluated in 59 healthy adults (32 women, 27 men; mean age 22.55 ± 3.79 years). The N75, P100, and N135 components, as well as the N75–P100 amplitude, were recorded with the application of two stimulus sizes (15′ and 60′). The statistical analysis revealed that the N75–P100 amplitude was significantly larger in women compared to men (*p* < 0.023), for both stimulus sizes and in all presentation conditions (monocular and binocular). Meanwhile, the latencies of the N75, P100, and N135 components did not differ significantly between the sexes. The authors emphasize that differences in amplitude may result from anatomical factors, such as smaller head size, and endocrine differences between the sexes, which influence the excitability of the visual cortex. The study’s conclusions indicate that sex primarily impacts the amplitude, not the latency, of the VEP waveform, confirming observations from previous studies and emphasizing the need to consider sex when establishing reference norms in electrophysiological studies.

In the study by Ekayanti et al. (2021) the impact of sex on VEP waveform parameters was assessed in a group of 120 healthy adults (60 women, 60 men) aged 18–65. The authors showed that the P100 amplitude was significantly greater in women than in men (for the left eye: 11.5 ± 5.1 μV vs. 8.0 ± 3.6 μV; for the right eye: 12.3 ± 5.9 μV vs. 8.3 ± 3.9 μV; *p* < 0.001), while the P100 latency did not differ significantly between the sexes. The authors explained the differences in amplitude by the smaller head circumference in women and the associated difference in brain volume. The possible involvement of hormonal factors was also suggested, though this requires further research.

In a study by Mirzaee Saba et al. (2023) differences in P100 wave parameters were analyzed in various areas of the visual field in 40 healthy adults (20 women, 20 men) aged 18–35 years (23.25 ± 3.44). The authors noted that there were no significant sex-related differences in P100 wave latency or amplitude in any of the examined visual field quadrants (*p* > 0.05). The obtained results indicate that in the young adult population, sex does not influence VEP pattern parameters, which confirms the stability of the physiological conduction properties and cortical responses in this area.

The study by Lek et al. (2019) involved 14 young adults aged 22–31 years (mean 27 ± 3 years) and 15 older adults aged 49–76 years (mean 63 ± 8 years). The authors analyzed the possible impact of sex on visual responses, however, found no significant differences between women and men in this regard. The analysis revealed no systematic deviations in any of the assessed electrophysiological parameters, indicating that sex did not in fact influence the nature of the recorded responses in this population.

In the study by Ura et al. (2021) the impact of biological factors, including sex, on VEP waveform parameters in 28 healthy adults aged 21–29 years (mean ± SD, 22.7 ± 1.7). The N75, P100, and N145 components, recorded using CRT and LCD monitors, were subjected to the study. The authors found no significant sex-related differences in the latency of any of the components, regardless of the monitor type. The results indicate that in the young adult population, sex does not influence in any meaningful way the time to VEP waveform, suggesting stability of visual pathway function during this period of life. The authors also note that the lack of a sex effect may be due to the narrow age range of the participants (21–29 years), thus, a group in which the influence of biological factors modulating latency may be minimal.

In the study by Thompson et al. (2023) VEP waveform parameters were assessed in a large group of 649 healthy children aged between 2 weeks and 16 years, from three centers using similar protocols consistent with ISCEV standards. The authors found that sex had no meaningful effect on P100 latency or amplitude, and the observed differences between girls and boys were minor, haphazard, and clinically insignificant. The only noticeable difference was noted in case of small stimulus fields (small check widths) and solely within the narrow infant age range of 8–18 weeks. In this group, girls demonstrated a slightly shorter P100 latency, although the difference was very small (< 10 ms) and within the range of natural developmental variability. Outside this age range and for large checkerboard squares, P100 latencies were comparable between the sexes. For this reason, the authors concluded that separate norms for girls and boys are unjustified, and that P100 development follows a similar course in both sexes.

In a study by Jiang et al. (2023) involving 101 healthy children aged 4 to 13 years, the development of pattern-evoked potentials (pattern VEP) was analyzed in the context of age, visual field, and sex. The authors found no significant sex-related differences in latency (N75, P100, and N135), amplitude, or amplitude difference (N75–P100, P100–N135). The results indicate that during childhood, from approximately 4 years of age to 13 years of age, pattern VEP parameters remain stable and comparable between girls and boys, suggesting that the functional maturation of the visual pathways in this respect is similar in both sexes.

The summary of available studies shows that the discrepancies regarding the effect of sex on pattern VEP parameters may not result solely from simple contradictions in the data, but rather from differences in the selection of the study population and the methodological conditions of registration. Studies which demonstrated significant sex-related differences typically concerned populations with a greater age diversity, used a wide range of stimulation parameters (various checkerboard square sizes, contrast, full-field and partial stimulation), or analyzed both quantitative and qualitative response parameters (wave morphology, PCA analysis). Under such conditions, subtle biological differences between women and men become more transparent, particularly in the amplitude of the P100 component and, in some studies, its latency.

A summary of the available studies indicates that the discrepancies in the effect of sex on VEP pattern parameters may result from differences in population selection (age, size, and sex distribution), the stimulation and recording parameters used, and the analysis methods adopted (e.g., amplitude and latency analysis vs. wave morphology assessment or PCA). In studies demonstrating significant sex differences, a larger P100 amplitude (or N75–P100) and sometimes shorter P100 latency were most frequently observed in women, whereas in studies with narrow age ranges or smaller sample sizes, the effect of sex was statistically elusive or clinically insignificant. In pediatric populations, most authors did not support the need for separate sex-specific norms, suggesting that potential sex differences are primarily revealed in adulthood and under conditions of greater variability in the studied material.

##### Refraction and visual acuity

Refraction and visual acuity are key factors determining the quality of recorded VEP pattern responses. Literature data clearly indicate that uncorrected refractive errors and reduced visual acuity lead to systematic changes in the electrophysiological recording, primarily including a longer P100 wave latency and a reduced amplitude.

Chen et al. (2023) assessed the relationship between visual acuity and P100 pattern VEP parameters in 24 healthy men (48 eyes) aged 20–35 years (mean 27.8 ± 4.3 years). Visual acuity was modified using lenses to levels of 1.0, 0.8, 0.6, and 0.4 (decimal values), and responses were recorded using six checkerboard element sizes (5.7°, 2.6°, 1°, 34′, 15′, and 7′). With a decrease in visual acuity, a significant increase in P100 latency and a decrease in its amplitude were observed. Both visual acuity and stimulus element size significantly influenced both parameters. A negative correlation between visual acuity and P100 latency was found for all pattern sizes, while a positive correlation between P100 amplitude and visual acuity was present for all sizes except 2.6° and 5.7°. The strongest correlations were obtained for the 1° stimulus, for which the correlation coefficient between visual acuity and P100 latency amounted to rs = −0.740, and between visual acuity and amplitude rs = 0.438. These results indicate that, at this stimulus size, P100 wave parameters most closely reflect changes in visual acuity resulting from purely refractive deterioration of retinal image quality.

Different conclusions were presented by Ekayanti et al. (2021) who assessed the effect of visual acuity on VEP pattern parameters in a population of 120 healthy adults. Individuals with visual acuity of at least 6/60, both with and without correction, were included in the analysis. More than half of the participants were myopic, but the authors did not specify the exact refraction values or standardize the full correction during VEP pattern recording. Despite this variability, no significant correlations were found between visual acuity and P100 wave latency or amplitude. These results suggest that in a healthy adult population, with small and unstandardized refractive differences, VEP pattern parameters may remain stable and independent of visual acuity.

The discrepancy between the results of experimental studies with controlled deterioration of retinal image quality and observations from normative studies may result from differences in refraction standardization, stimulus selection, and the range of variability in visual acuity in the study population.

The effect of controlled refractive blur on the pattern VEP response was analyzed in detail by Kordek et al. (2022) The authors demonstrated that gradually increased defocus leads to clear, quantitative changes in pattern VEP parameters, the nature of which depended on the size of the pattern elements. The amplitude of the response systematically decreased with the increasing optical blur, whilst the dynamics of this decrease is significantly greater for small checkerboard squares. For small squares, the amplitude decreased on average by 2.6 μV for each added dioptre, while for larger squares, the decrease was approximately 1.2 μV per dioptre. At the same time, an increase in P100 latency was observed, also dependent on the stimulus size: for small fields, the latency increased by approximately 15 ms per dioptre of blur, while for large fields, this rate was significantly slower, reaching approximately 3 ms per dioptre. These results indicate that degrading the retinal image quality through controlled optical blur simultaneously leads to the weakening of the pattern VEP response and the slow-down of P100 wave generation.

The importance of retinal image quality for pattern VEP parameters was also confirmed by Yang et al. (2019). They conducted a study assessing the effect of higher-order aberrations (HOAs) on the pattern-reversal VEP response. The authors indicate that optical aberrations are a significant factor influencing the quality of the VEP recording, as they degrade the quality of the retinal image of the stimulus. The study compared pattern-reversal VEP recordings obtained after correction of only low-order aberrations (LOA: defocus and astigmatism) with recordings after full correction of aberrations up to the 8th order using an adaptive optics system. Higher-order aberrations were shown to significantly affect the amplitudes of all pattern-reversal VEP components. After HOA correction, an increase in N1 amplitude was observed, ranging from 15 to 59%, depending on the spatial frequency, with maximum values occurring at 4 cycles per degree (cpd). P1 amplitude increased by 9 to 36%, reaching its highest values at 1 and 16 cpd, while N2 amplitude increased by 3 to 32%, with a maximum at 16 cpd. These changes had varying statistical significance—increases in P1 amplitude (except for 2 cpd) and N2 amplitude (for 12 and 16 cpd) were statistically significant. Notwithstanding the above, no statistically significant effect of HOAs on N1, P1, or N2 latencies was found. Based on the comparison of recordings before and after HOA correction, the authors emphasize that ophthalmic aberrations—especially higher-order aberrations—differentiate the amplitudes and latencies of individual pattern-reversal VEP components (N1, P1, and N2). Aberration correction improved the retinal image quality, which allowed for stimulation of a larger number of retinal cells, which, in turn, received a clearer image, translating into an increase in the amplitudes of the measured components. The authors reiterated that the lack of significant changes in latencies may be due to the fact that stimulation parameters, such as spatial frequency and stimulus field size, were not changed after HOA correction. The authors of the study also noted that the amplitudes of pattern-reversal VEP components reflect the quality of the retinal stimulus representation, supporting the possibility of using this test for the objective assessment of refractive power and visual function.

In a study by Baumgarten et al. (2022), the effect of pseudophakia on full-field pattern VEP parameters was assessed. The analysis conducted in a group of individuals over 60 years of age showed that the presence of an artificial lens was not associated with any significant changes in the latency or amplitude of key response components, including the N75, P100, and N135, regardless of the size of the checkerboard. These results show that pseudophakia, under conditions of normal visual system function, does not affect the pattern VEP response, and the recorded signals remain comparable to those obtained in eyes with a natural lens.

In summary, refraction and visual acuity significantly modulate pattern VEP parameters. Uncorrected refractive defects and reduced retinal image quality lead to prolonged P100 latency and reduced response amplitude, with the nature and severity of these changes depending on the size of the pattern elements and the degree of optical blur. Studies with controlled dioptric defocus indicate that changes in pattern VEP parameters increase systematically with an increasing blur, and this effect is particularly pronounced for stimuli with high spatial frequency, i.e., small checkerboard squares. The available data, including experimental results with the use of pattern-reversal, indicate that pattern VEP is highly sensitive to even subtle deterioration in retinal image quality, which can manifest as measurable changes in P100 wave amplitude (Chenguiti et al., 2023). At the same time, normative studies conducted in healthy adult populations confirm that small, non-standardized refractive differences and preserved visual acuity may not significantly affect pattern VEP parameters. Taking into consideration the refraction, visual acuity, and optical aberrations remains crucial for the correct interpretation of pattern VEP results, especially in clinical trials and when comparing data between centers using different recording protocols consistent with current ISCEV recommendations.

##### Ocular dominance

Ocular dominance has been the subject of analyses in several electrophysiological studies, however, its influence on the VEP pattern response parameters remains ambiguous and is usually subtle. In the subject literature, the concept of ocular dominance is often understood as both the functional advantage of one eye in visual tasks and the interocular asymmetry of electrophysiological response parameters, which makes direct comparisons of individual studies challenging.

Willeford et al. (2016) evaluated the feasibility of using the VEP pattern as an objective tool for determining ocular dominance. In ten healthy adults aged 50–70 years, responses were recorded upon binocular stimulation, introducing monocular defocus of the dominant and non-dominant eye (+1 D, +2 D, and +3 D). Applying defocus to either eye led to a typical pattern VEP response, including a reduction in amplitude and a prolongation of the P100 latency, which was observed in 92% of the measurement conditions. However, the key finding of the study was the lack of significant differences in response parameters between defocusing the dominant and non-dominant eyes. The authors concluded that the pattern VEP is not sensitive to ocular dominance and, therefore, is not a useful tool for objectively determining dominance or assisting in the selection of monovision.

Similar conclusions were drawn pursuant to the conduct of normative studies that analyzed the symmetry of the pattern VEP response between the right and left eye. Mahjoob et al. (2019) assessed pattern VEP responses in 59 healthy adults (mean age 22.6 ± 3.8 years). The authors found no significant interocular differences in the latencies of the N75, P100, and N135 components, nor in the N75–P100 amplitude. Minor differences presented in the study were physiological in nature and were proposed as reference values, enabling the distinction between normal and pathological changes. The authors considered any difference exceeding these ranges to be a potential warning sign, requiring further ophthalmological or neurological diagnostics.

Similar observations were reported by Ekayanti et al. (2021) study of 120 healthy individuals aged 18–65 years. The authors presented separate values of the VEP pattern parameters for the left and right eye, demonstrating very high response symmetry. The average P100 latency was 104.6 ± 3.4 ms for the left eye and 104.1 ± 3.4 ms for the right eye, while the N75–P100 amplitude reached 9.8 ± 4.7 μV and 10.3 ± 5.4 μV, respectively. Despite the lack of formal statistical analysis comparing both eyes, these differences remained within the range of physiological variability and did not indicate a systematic advantage of one eye. The achieved results confirm the high symmetry of the VEP response pattern in the adult population.

No significant interocular differences were also noted in the paediatric population. Jiang et al. (2023) analyzed pattern VEP responses in 101 healthy children aged 4–13 years and found no significant differences between the right and left eye in the latencies of the N75, P100, and N135 components or in the response amplitudes. The authors emphasized that ‘interocular vision’ did not influence the course of response which proves a high physiological symmetry of pattern VEP in this age group.

Somewhat different results were presented by Utrobičić et al. (2023) in the study focused on analysing pattern VEP responses in healthy children serving as the reference group. In this study, the dominant eye was defined as the eye exhibiting a shorter latency and/or a larger P100 amplitude. The authors detected interocular asymmetry, with the dominant eye being characterized by a shorter P100 latency (median difference of approximately 1.2 ms) and a larger P100 amplitude (on average by 1.5 ± 0.6 μV). It has also been established that latency asymmetry decreases with age, while the amplitude advantage of the dominant eye persists, although its magnitude gradually decreases. However, it ought to be emphasized that in this study, ocular dominance was defined directly based on pattern VEP parameters, which limits the possibility of independent interpretation of the observed differences and suggests that the described asymmetries reflect physiological interocular variability rather than classically understood ocular dominance.

Most studies have failed to confirm the existence of a meaningful, systematic effect of ocular dominance on pattern VEP response parameters. In both adult and paediatric populations, these responses are distinguished by high interocular symmetry, and the observed differences typically fall within the limits of physiological variability. Some studies have described minor interocular asymmetries, which have been interpreted as a manifestation of ocular dominance. Nevertheless, their significance appears limited and strongly dependent on the adopted methodology and definition of dominance. All in all, ocular dominance does not play a significant role in the routine interpretation of pattern VEP results, and its potential importance may be of an ancillary nature, primarily in research or algorithmic analyses, rather than in standard clinical diagnostics.

#### Anthropometric factors

##### Body weight and body mass index (BMI)

Some studies concerning populations without overt neurological or ophthalmological diseases have demonstrated a positive relationship between body mass index (BMI) and P100 wave latency and a negative relationship between BMI and P100 amplitude. However, the observed effects were usually small and did not always reach statistical significance (Ekayanti et al., 2021). Sharma et al. (2015) found a substantial positive correlation between body weight, BMI, and body surface area (BSA) and P100 latency in women, with no similar relationship in men.

In paediatric populations including overweight and obese children, thus, groups at increased metabolic risk, prolonged P100 latency was observed compared to children with normal weight. Additionally, a comparison of children with obesity and co-existing insulin resistance with obese children without carbohydrate metabolism disorders revealed a more pronounced prolonged P100 latency in the former group. The authors attribute this effect to hyperglycaemia and early, subclinical changes in retinal cells, which may precede the development of retinopathy (Fort et al., 2011).

Similar observations were reported by Korkmaz et al. (2019) demonstrated a significantly prolonged P100 latency in patients with impaired glucose tolerance. The authors suggest that the VEP pattern test may be of a prognostic value in assessing individuals with prediabetes who exhibit retinal changes similar to diabetic retinopathy. This indicates that carbohydrate metabolism disorders, such as insulin resistance or glucose intolerance, may affect the function of retinal cells, including ganglion cells, as reflected in VEP pattern parameters, particularly the prolonged P100 latency.

Gupta et al. (2017) interpreted the observed prolonged P100 latency in overweight and obese individuals as a potential effect of chronic, low-grade inflammation. The authors attribute this to the fact that obesity is a state of chronic, low-grade inflammation accompanied by tissue hypoxia, contributing to an increased secretion of proinflammatory cytokines such as interleukin-1, interleukin-6, and tumour necrosis factor alpha (TNF-*α*). Increased cytokine concentrations increase oxidative stress and the production of free radicals, which can lead to damaging nerve cells, including Schwann cells responsible for myelinogenesis. Therefore, the myelin structural abnormalities observed in obesity may contribute to slower nerve impulse conduction.

Different results were presented by Narra et al. (2023), who found neither significant differences in P100 latency between young obese men and those with a correct body weight, nor a correlation between P100 latency and BMI. The discrepancies between the study results indicate that the effect of body weight and BMI on VEP pattern parameters is not clear-cut and likely becomes apparent only in the presence of metabolic disorders such as insulin resistance or impaired glucose tolerance. In young adult populations without dysmetabolism features, pattern VEP parameters typically remain stable, while in overweight and obese groups, a tendency toward longer P100 latencies is observed, which may reflect early, subclinical impairments in retinal function and nerve conduction.

##### Head circumference and size

Most studies published in the last decade, conducted using standardized pattern VEP recording protocols, do not confirm a significant effect of head structural parameters (such as head circumference or length) on P100 wave amplitude or latency, in either women or men (Ekayanti et al., 2021; Sharma et al., 2015).

Older publications, however, indicated a relationship between head circumference and P100 latency, while no significant association was detected with respect to head length (Gregori et al., 2006; Guthkelch et al., 1987; Solanki et al., 2013). The authors of these studies suggested that the shorter P100 latency observed in women may result from differences in head geometry and size, rather than from biological differences related to sex. This interpretation has been widely cited as a potential explanation for sex differences in pattern VEP parameters.

However, in light of more recent normative studies, this hypothesis has limited clinical significance, and the effect of head size on pattern VEP parameters is currently considered secondary to other biological and methodological factors.

##### Height

Some authors do not confirm the existence of a significant relationship between height and the latency or amplitude of the P100 wave in healthy populations. Kothari et al. (2013) however, demonstrated a positive correlation between height and the latencies of the N70, P100, and N155 components, and a negative correlation between height and the P100 amplitude. The authors interpreted these observations as a potential effect of the greater distance between the retina and the cortical generators of visual responses in taller individuals, located within the primary visual cortex (Brodmann area 17) near the carina sulcus of the occipital lobe.

Morphometric data also indicate that taller individuals are characterized by larger skull dimensions and longer axial eye length. (Allison et al., 1983) Larsen (1979) demonstrated a correlation between axial eye length and head circumference, head dimensions, and height, as well as differences in mean eye length between women and men, with higher values observed in men. The correlations observed in older studies between height or head size and VEP pattern parameters should be interpreted in the context of the recording conditions at the time and the lack of full standardization of stimulation and signal acquisition parameters, which could have increased the influence of geometric and anthropometric factors on the obtained results.

However, in light of the available data, the effect of height on VEP pattern parameters appears indirect and non-specific, and the observed correlations may result from the association of height with other anthropometric characteristics, such as head size or axial eye length, rather than from a direct effect on the function of the visual pathways.

##### Race

According to a single study, Caucasian individuals have longer P100 latencies compared to Black individuals, in both women and men (de Graaf et al., 1985). It should be emphasized that most studies examining ethnic differences in VEP parameters were conducted before the introduction of unified recording standards. Hence, the observed differences should be interpreted with caution, considering them an indirect effect of anthropometric and methodological differences rather than a direct influence of ethnicity. Current research focuses primarily on the local validation of reference standards, in accordance with ISCEV recommendations, which may explain the lack of more recent reports in this area.

#### Environmental and exposure factors

The VEP response pattern parameters may be modulated by environmental and exposure factors, although research results in this area are inconsistent and often depend on the exposure dose, age of the subjects, and technical recording conditions. The subject literature describes factors with a significant effect on the latency and amplitude of the main response components, as well as factors that did not significantly change the VEP pattern parameters.

##### Alcohol

Kim et al. (2016) assessed the acute effect of ethanol on VEP pattern parameters in healthy adults. Responses were recorded before alcohol consumption and after oral administration of ethanol at a dose of 0.75 g/kg body weight. Average blood alcohol concentration at the time of testing was 0.034% ± 0.05%. The most pronounced effect was a significant prolongation of the P100 latency, which increased from 109.4 ± 5.3 ms to 113.1 ± 8.2 ms (*p* = 0.008). No significant changes were found in the latencies of the N75 and N135 components or in the N75–P100 and P100–N135 amplitudes. These results indicate that alcohol consumption leads to a slow-down of visual processing at the cortical level, without affecting the magnitude of the VEP pattern response.

##### Caffeine

In a randomized, controlled study, Top Karti et al. (2019) evaluated the acute effect of caffeine consumption on VEP pattern parameters. Participants received a single dose of approximately 216 mg of caffeine or placebo, and recordings were performed before and after 1 hour of drinking. The authors found no significant changes in P100 latency or amplitude after caffeine consumption (*p* > 0.05). These results suggest that a moderate dose of caffeine does not significantly affect VEP pattern parameters, and routinely restricting caffeine intake before testing is not necessary.

##### Dietary factors in early life

The influence of infant feeding on the development of visual responses was assessed by means of a randomized COGNIS study (Nieto-Ruiz et al., 2019). Nieto-Ruiz et al. demonstrated that infants fed both with a standard formula and an enriched formula with bioactive ingredients were characterized by longer latencies and lower P100 amplitudes compared to breastfed infants. No significant differences were found between the two formulas in the standard P100 parameters. However, the authors noted that in the group fed by means of the enriched formula, the percentage of infants responding to the smallest stimulus angle (7.5′) increased at 12 months of age, reaching values similar to those observed in breastfed infants. These results suggest that early feeding patterns are reflected in VEP patterns and should be considered when interpreting results from studies in infants.

##### Exposure to electromagnetic fields

The effects of electromagnetic fields emitted by mobile phones were analyzed in a study examining their effect on VEP pattern latency (Singh, 2016). The authors demonstrated that short-term exposure to electromagnetic fields was associated with prolonged P100 latencies compared to control conditions, with no significant changes in response amplitude. The observed effect was interpreted as a potential modulation of visual cortex excitability, but the authors emphasized its preliminary nature and lack of clear clinical significance.

##### Prenatal factors

The PREOBE study (Torres-Espínola et al., 2018) assessed the influence of pregnancy on VEP pattern parameters in offspring. It was demonstrated that children born to mothers with gestational diabetes had significantly prolonged P100 latencies at 18 months of age, particularly for smaller stimulus angles. This effect was the most explicit in cases of gestational diabetes coexisting with maternal overweight or obesity, whereas overweight or obesity alone without diabetes did not cause significant changes in pattern VEP parameters. These results indicate that information regarding the course of pregnancy, including the presence of gestational diabetes and maternal body weight, may be important when interpreting pattern VEP responses in infants. These observations suggest that prenatal factors may modulate the early functional maturation of the visual pathways, which is reflected in the cortical response parameters assessed using pattern VEP.

##### Physiological status of the test subject

The effect of sleep deprivation on pattern VEP parameters was analyzed by Dziadkowiak and Podemski (2019). The authors found no significant changes in the control group in response latency or amplitude after sleep deprivation compared to baseline values. These results suggest that in healthy individuals, short-term sleep deprivation does not necessarily significantly affect pattern VEP parameters.

#### Predisposing factors—family history of migraine

Studies examining the influence of family history of migraine have shown (Lisicki et al., 2017) that healthy individuals with a positive family history are characterized by a different pattern VEP response profile. They exhibited a deficit in visual habituation and reduced N1-P1 amplitude in the initial blocks of stimulation compared to individuals without a family history, with no latency changes. The nature of these changes was similar to that observed in migraine patients during the interictal period, indicating that genetic predisposition may influence the functional organization of cortical responses assessed using pattern VEP.

### Summary

Environmental and exposure factors may modulate pattern VEP parameters to varying degrees. Alcohol leads to a prolongation of the P100 latency, while moderate doses of caffeine have no significant effect on the response. Infant feeding habits and prenatal factors, such as gestational diabetes, are reflected in pattern VEP parameters, highlighting the importance of interview data in interpreting test results in children. Exposure to electromagnetic fields and genetic predispositions, including a family history of migraine, can modify the selected features of cortical responses. At the same time, not all physiological factors, such as sleep deprivation in healthy individuals, reveal meaningful pattern VEP parameters. The obtained data emphasize the need to control environmental conditions and take into account the patient’s condition in order to obtain reliable and comparable pattern VEP recordings.

A detailed summary of studies presented in this review is provided in Supplementary Tables S1–S2. In particular, Supplementary Table S1 includes the characteristics of the studied populations, stimulus parameters, recording parameters, and the direction of observed changes in P100 latency and amplitude, whereas Supplementary Table S2 presents corresponding information for excluded studies together with the reasons for exclusion.

## Discussion

### Synthesis of evidence

**Figure 2:**
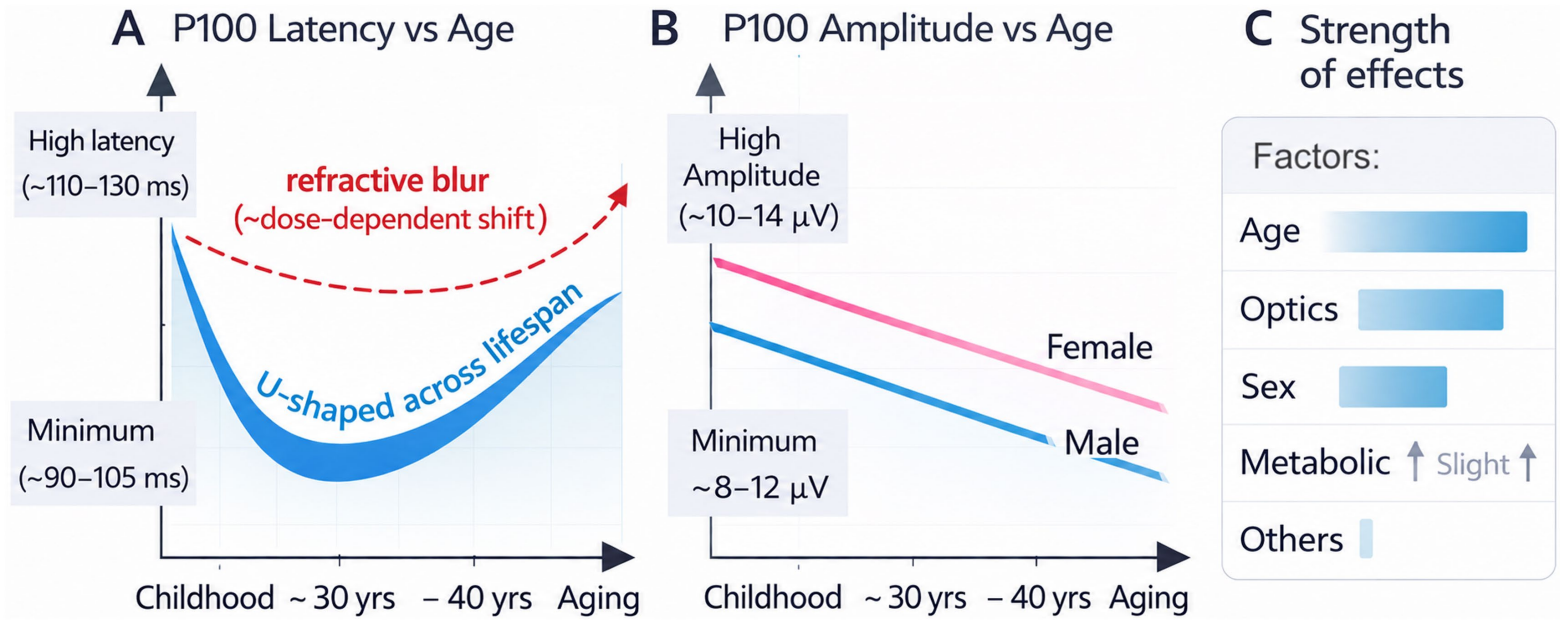
Schematic summary of the strongest evidence-based determinants of P100 parameters. **(A)** Age-related changes in P100 latency across the lifespan, illustrating a non-linear (U-shaped) trajectory with shortening during maturation, relative stabilization in early adulthood, and prolongation with aging; the effect of refractive blur is shown schematically as a dose-dependent latency shift. **(B)** Age and sex-related trends in P100 amplitude, showing a general decline with aging and consistently higher amplitudes in females compared with males. **(C)** Relative strength of effects influencing P100.

The variability of normative P100 values across studies, as summarized in Table 1, provides an important context for interpreting the overall direction and strength of the reported effects, which are summarized in Table 2, whereas the most robust and consistently observed relationships are illustrated schematically in Figure 2.

**Table 2:** Direction of effects of individual factors on P100 latency and amplitude in pattern VEP (2015–2025).

Factor	P100 latency	P100 amplitude	Consistency	Strength of evidence
Age	↑ (after ~40 yrs); U-shaped across lifespan	↓ with aging	High	Strong
Sex	↔/slight ↓ in women	↑ in women (adults)	Moderate	Moderate
Refractive blur	↑ (dose-dependent)	↓ (dose-dependent)	High	Strong
Higher-order aberrations	↔	↑ after correction	Moderate	Moderate
Ocular dominance	↔	↔	High (no systematic effect)	Weak
BMI / metabolic disturbance	↑ (mainly dysmetabolic groups)	↓ (in some studies)	Low–moderate	Limited
Head size	↔	↔	Inconsistent	Weak
Height	↑ (possible)	↓ (possible)	Low	Weak
Alcohol (acute)	↑	↔	Single study	Limited
Caffeine (acute)	↔	↔	Consistent null effect	Moderate
Prenatal factors (GDM)	↑ (infancy)	↓ (infancy)	Limited but consistent	Moderate

↑, increase; ↓, decrease; ↔, no consistent or clinically meaningful effect.

Direction refers to the relationship between the analyzed factor and the parameter value as reported in the original studies.

### Key findings

#### Age is the most robust and consistently reported determinant of P100 parameters

Across the lifespan, P100 latency follows a non-linear trajectory, with progressive shortening during maturation, stabilization in early adulthood (around the third decade), and gradual prolongation after approximately 40 years of age. P100 amplitude generally decreases with aging, particularly in later decades of life.

#### Sex differences predominantly affect amplitude rather than latency

In most adult populations, women demonstrate higher P100 (or N75–P100) amplitudes and, in some studies, slightly shorter latencies. However, these differences are less consistent in paediatric or narrow-age cohorts.

#### Retinal image quality exerts a strong, dose-dependent effect on P100 parameters

Controlled refractive blur and higher-order aberrations systematically prolong P100 latency and reduce amplitude. These effects are particularly pronounced for small check sizes and high spatial frequencies.

#### Ocular dominance does not produce clinically meaningful asymmetry in pattern VEP recordings

Interocular differences in latency and amplitude remain within physiological variability in both adult and paediatric populations, and most studies do not support a systematic dominance-related effect.Metabolic and anthropometric factors show conditional or weak effects

Increased BMI and metabolic disturbances (e.g., insulin resistance or impaired glucose tolerance) may be associated with prolonged P100 latency, particularly in paediatric or dysmetabolic populations. In contrast, head size, height, and other anthropometric variables demonstrate inconsistent or minimal associations in recent standardized studies.

#### Environmental exposures exert selective effects

Acute alcohol intake prolongs P100 latency, whereas moderate caffeine consumption does not significantly influence latency or amplitude. Prenatal metabolic conditions, such as gestational diabetes, may affect early-life VEP maturation.

#### Compared to age and optical factors, most other modifiers exert modest effects

Age and retinal image quality emerge as the primary physiological determinants of P100 latency and amplitude in healthy individuals.

The VEP pattern test is a sensitive and valuable tool in the functional assessment of the visual pathways. However, correct interpretation of the obtained results requires consideration of numerous biological, refractive, anthropometric, and environmental factors that can subtly but measurably modulate the response parameters. The variability of normative values observed across studies (Table 1) indicates that universal reference standards are of limited practical value unless they are grounded in the specific technical and organizational conditions of a given laboratory.

### Limitations

Our work is not deprived of any limitations. One of these is the heterogeneity of the analyzed studies, resulting from differences in equipment, stimulation parameters, and signal analysis methods, which prevents quantitative meta-analysis. Importantly, differences in stimulation and recording parameters may substantially influence pattern VEP outcomes and may, in some settings, have a greater impact than the physiological characteristics of the examined subjects, thereby partially explaining inconsistencies across studies. In addition, intraindividual variability and response reproducibility were not analysed separately in this review, as these aspects were not consistently reported across the included studies. Yet another limitation concerns the fact that the review is based primarily on observational studies, often with small sample sizes or covering narrow age ranges. Moreover, some of the analysed studies do not fully report confounding factors, such as refraction or recording conditions. Finally, the review focuses on healthy populations, meaning that the findings cannot always be directly extrapolated to groups of patients with ophthalmological or neurological diseases, in whom the influence of individual factors may be modified by the pathological process. As a result, the conclusions are synthesized and qualitative in nature, and their direct translation to clinical populations should be made with caution.

An additional limitation of this study is the necessity to refer to the selected publications published before 2015. These studies were not included in the comparative analysis or qualitative synthesis of the results, serving solely as contextual information, providing a framework for presenting the historical research foundation and primary observations regarding the physiological variability of the VEP pattern response. Their interpretation was conducted taking into account the methodological differences and the fact that they were developed before the introduction of current ISCEV standards, which limits the possibility of direct comparison of their results with contemporary data.

One of the main advantages of this study is the comprehensive and multidimensional approach to individual factors influencing VEP pattern parameters, including biological, anthropometric, refractive, environmental, and exposure variables. Unlike many previous studies, which focused on single determinants (most often age or sex), this review summarizes a broad spectrum of factors that can modify both the latency and amplitude of the main components of the VEP response. Another strength of the study is that the analysis is grounded in current ISCEV standards, taking into account technological and methodological changes from 2005 to 2026. This allows for a critical interpretation of literature results in the context of changing recording conditions (monitor types, stimulation parameters, acquisition protocols), which has direct practical implications for electrophysiology laboratories. The large number of analysed publications and the consistent selection of studies meeting substantive and methodological criteria are also noteworthy. This allowed us to identify reproducible physiological trends (e.g., the relationship between age and sex with P100 latency and amplitude), as well as to pinpoint areas that are ambiguous or less well-documented, such as the influence of ocular dominance or environmental factors. An additional value of this work is the integration of electrophysiological data with neuroanatomical and physiological aspects, which allows for a better understanding of the mechanisms underlying the observed changes in VEP patterns. This approach facilitates the interpretation of results not only in normative but also pathophysiological terms.

## Conclusion

Thus, each centre specializing in visual electrophysiology should develop its own standards for VEP pattern recording and interpretation, taking into account the equipment used, stimulation parameters, study population, and environmental conditions. These standards should be developed and reported as closely as possible to ISCEV standards, enabling comparability between centres and ensuring that the obtained results can be interpreted unambiguously by other physicians and researchers involved in ocular electrophysiological diagnostics.

This approach not only increases the diagnostic reliability of the VEP pattern in clinical practice but also the value of this method in research and multi-centre analyses, enabling more precise differentiation between physiological and pathological changes and improving interpretative consistency between different electrophysiology laboratories.
