# Correction: Physiological determinants of cortical P100 responses in pattern visual evoked potentials: a scoping review

**DOI:** 10.3389/fnins.2026.1892090

**Published:** 2026-06-18

**Authors:** Łukasz Lisowski, Jolanta Lisowska, Łukasz Łabieniec, Mateusz Zarzecki, Dominik Zalewski, Iwona Obuchowska, Joanna Konopińska

**Affiliations:** 1 Department of Ophthalmology, Medical University of Bialystok, Bialystok, Poland; 2 Individual Medical Practice, Bialystok, Poland; 3 Faculty of Physics, University of Bialystok, Bialystok, Poland; 4 Lens Center for Eye Diagnostics and Microsurgery, Olsztyn, Poland

**Keywords:** aging brain, cortical processing, ISCEV standards, P100 amplitude, P100 latency, pattern visual evoked potential, retinal image quality, visual electrophysiology

There was a mistake in Figure 2 as published. In panel C, the label “Optics” was incorrectly displayed as “Opglalte.” The corrected Figure 2 appears below.

**Figure 2:**
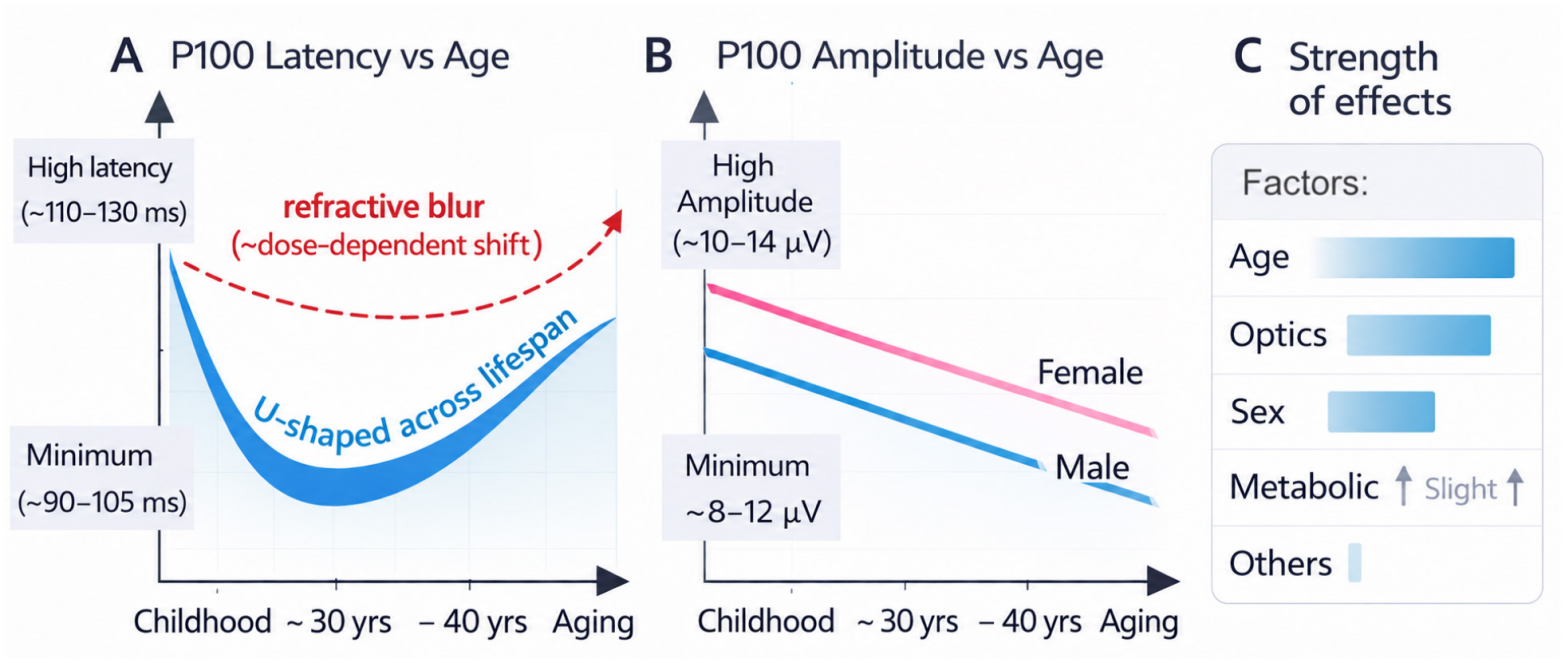
Schematic summary of the strongest evidence-based determinants of P100 parameters. **(A)** Age-related changes in P100 latency across the lifespan, illustrating a non-linear (U-shaped) trajectory with shortening during maturation, relative stabilization in early adulthood, and prolongation with aging; the effect of refractive blur is shown schematically as a dose-dependent latency shift. **(B)** Age and sex-related trends in P100 amplitude, showing a general decline with aging and consistently higher amplitudes in females compared with males. **(C)** Relative strength of effects influencing P100.

The original version of this article has been updated.

