# Supplementary material for: Physiological determinants of cortical P100 responses in pattern visual evoked potentials: a scoping review
Source: Front Neurosci. 2026 May 26;20:1821657. doi: 10.3389/fnins.2026.1821657 (PMC13246599; doi:10.3389/fnins.2026.1821657)
Supplement: Supplementary file 1 [file Table_1.docx]

| **Author (year)** | **Title** | **Population** | **Stimulus parameters** | **Recording parameters** | **Direction of effects** | **Main conclusions (authors)** |
| --- | --- | --- | --- | --- | --- | --- |
| **Kim et al. (2016)** | The Effects of Alcohol on Visual Evoked Potential and Multifocal Electroretinography | Healthy adults; n=15; mean age ~31 years; Korea | Pattern-reversal checkerboard; check size 0.25°; contrast ~99%; mean luminance <1 cd/m² (black) and 115 cd/m² (white); monocular stimulation; viewing distance 1 m; reversal rate 1 rps | Oz–Fz derivation (10–20); ground forehead; Roland-Consult RetiScan; band-pass not specified; 100 sweeps averaged; repeated recordings until stable waveform | P100 latency ↑ after alcohol administration; P100 amplitude ↔ | P100 latency increased significantly after acute alcohol intake, while P100 amplitude showed no significant change |
| **Benedek et al. (2017)** | The Development and Aging of the Magnocellular and Parvocellular Visual Pathways as Indicated by VEP Recordings between 5 and 84 Years of Age | Healthy children and adults, 5–84 years; n=115; Hungary | Pattern reversal checkerboard; check sizes 15′ (0.25°) and 60′ (1°); achromatic contrast 97% (plus low-contrast and chromatic conditions); mean luminance ~100 cd/m²; monocular stimulation; reversal rate ~0.9 Hz | Oz–Fz derivation (10–20); ground on forehead; band-pass 1–100 Hz; 100 sweeps averaged; clinical VEP system (Roland Consult) | P100 latency ↓ with age until young adulthood, then ↑ with aging; P100 amplitude ↓ with age | P100 latency shortens during development and prolongs with aging, while P100 amplitude decreases progressively across the lifespan |
| **Sawaya et al. (2017)** | Pattern reversal visual evoked potentials in adults: variability with age | Healthy adults, 20–92 years; n=81 (162 eyes); Canada | Pattern reversal checkerboard; check size 1°; high-contrast black–white stimulus; mean luminance ~50 cd/m²; monocular stimulation; reversal rate 2 Hz | Oz–Fz derivation (10–20); ground forehead; band-pass 1–100 Hz; sweep 250 ms; 150 sweeps averaged; clinical VEP system | P100 latency ↑ with age (non-linear); P100 amplitude ↓ with age | P100 latency shows age-related prolongation with inter-individual variability, while P100 amplitude decreases progressively with increasing age |
| **Korkmaz et al. (2018)** | Evaluation of visual pathways using visual evoked potentials in the patients with impaired fasting glucose and impaired glucose tolerance | Patients with impaired fasting glucose, impaired glucose tolerance, and healthy controls; adults; Turkey | Pattern reversal checkerboard; check size ~23′; contrast 100%; mean luminance ~50 cd/m²; monocular stimulation; reversal rate 2 rps | Oz derivation (10–20); reference at vertex; band-pass 1–100 Hz; sweep 300 ms; 128 sweeps averaged | P100 latency ↑ in impaired fasting glucose and impaired glucose tolerance vs controls | P100 latencies were significantly prolonged in patients with impaired fasting glucose and impaired glucose tolerance compared with healthy controls |
| **Mermeklieva (2018)** | Reference values of pattern reversal visual evoked potentials in Bulgarian population | Healthy adults, 27–51 years; n=47; Bulgaria | Pattern reversal checkerboard; check sizes 0.25° (15′) and 1° (60′); high-contrast black–white stimulus; monocular stimulation; reversal rate 1 Hz (2 rps) | Oz–Fz derivation (10–20) with additional O1/O2; band-pass 1–100 Hz; ≥100 sweeps averaged; reproducible recordings | P100 latency ↑ with age; P100 amplitude ↔ with age | P100 latency increased with age, while P100 amplitude showed no significant age-related changes |
| **Yang et al. (2019)** | Effect of high-order aberrations on pattern-reversal visual evoked potentials | Healthy adults, 20–32 years; n=12; China | Pattern reversal sinusoidal grating; central field size 2°; spatial frequencies 1–16 cpd; contrast >95%; background luminance ~4.7 cd/m²; monocular stimulation; reversal rate 4 rps; adaptive optics correction of high-order aberrations | Oz derivation (10–20); reference on forehead; ground earlobe; band-pass 1–30 Hz; sampling rate 1000 Hz; 100 sweeps averaged; three repetitions | P100 amplitude ↑ with HOA correction; P100 latency ↔ with HOA correction | P100 (P1) amplitude increased significantly after correction of high-order aberrations, while P100 latency showed no consistent or significant changes |
| **Lek et al. (2019)** | An Electrophysiological Comparison of Contrast Response Functions in Younger and Older Adults, and Those With Glaucoma | Healthy younger adults, healthy older adults, and adults with early glaucoma; n=63; Australia | Pattern reversal checkerboard; check size ~0.88°; contrast levels 4–97%; mean luminance ~50 cd/m²; monocular stimulation; steady-state pattern reversal at high temporal frequency | Electrodes placed around Oz with reference at Fz; band-pass ~1–100 Hz; steady-state PVEP recorded simultaneously with PERG; frequency-domain analysis | PVEP amplitude ↓ with age and glaucoma; PVEP latency ↔ between groups | PVEP contrast response amplitude was reduced in older adults and in participants with early glaucoma compared with younger controls, while PVEP phase/latency did not differ significantly between groups |
| **Top Karti et al. (2019)** | Acute Effect of Caffeine on Pattern-Reversal Visual Evoked Potential: A Randomized-Controlled Study | Healthy adults; n=40 (20 caffeine, 20 control); 21–59 years; Turkey | Pattern-reversal checkerboard; check size ~37′; black–white stimulus; monocular full-field stimulation; reversal rate 1 Hz; refractive correction during testing | Oz–Cz derivation (10–20); ground forearm; band-pass 2–100 Hz; analysis time 500 ms; 256 sweeps averaged | P100 latency ↔ after caffeine intake; P100 amplitude ↔ after caffeine intake | Moderate acute caffeine intake did not produce significant changes in P100 latency or amplitude compared with baseline or placebo |
| **Dahanayake et al. (2020)** | Normal values of pattern reversal visual evoked potentials (PRVEP) and pattern electroretinography (PERG) in healthy adults in Sri Lanka | Healthy adults, 20–62 years; n=50 (11 males, 39 females); Sri Lanka | Pattern reversal checkerboard; check sizes 1° and 0.25°; contrast ≥80% (Michelson); mean luminance 50 cd/m²; CRT display; monocular stimulation; reversal rate 2 Hz | Oz–Fz derivation (10–20); ground Cz; band-pass 1–100 Hz; 100 sweeps averaged; two replications | P100 latency ↔ with sex; P100 amplitude ↔ with sex | No significant differences in P100 latency or amplitude were found between males and females |
| **Ekayanti et al. (2021)** | Normative values of visual evoked potential in adults | Healthy adults, 18–65 years; n=120 (60 male, 60 female); Indonesia | Pattern reversal checkerboard; check size 26′; contrast 80%; viewing distance 1 m; monocular stimulation; room lighting 60 lx | Oz–Fz (10–20); band-pass 1–100 Hz; analysis time 250 ms; 200 sweeps averaged; two recordings | P100 latency ↔ with age and sex; P100 amplitude ↓ with age; P100 amplitude ↑ in females | Age and sex do not affect P100 latency; P100 amplitude decreases with age and is higher in females; anthropometric factors show no significant association |
| **Patterson Gentile et al. (2021)** | Developmental Effects on Pattern Visual Evoked Potentials Characterized by Principal Component Analysis | Children, adolescents and adults; 6–44 years; n=116; USA | Pattern reversal checkerboard; check size ~1° (ISCEV large checks); contrast 97%; mean luminance ~50 cd/m²; binocular stimulation; reversal rate 2 rps | Oz–Fz derivation (10–20); sampling rate 1024 Hz; notch filtering at 60/120 Hz; baseline correction; five recording blocks | P100 latency ↓ with age; P100 amplitude ↔ with age | P100 latency decreases with age, while P100 amplitude does not show significant age-related changes |
| **Jiang et al. (2021)** | Developmental characteristics of visual evoked potentials to different stimulation in normal children | Healthy children, 4–13 years; n=101 (PVEP group); China | Pattern reversal checkerboard; check size 1° (60′); Michelson contrast 95%; mean luminance 50 cd/m²; viewing distance 100 cm; monocular and binocular stimulation; reversal rate 2 Hz | Oz–Fz (10–20); band-pass 1–70 Hz; ≥100 sweeps averaged; ≥2 reproducible recordings | P100 latency ↑ with age; P100 amplitude ↔ with age | PVEP latency showed a weak but significant age-related prolongation, while P100 amplitude did not change significantly with age |
| **Ura et al. (2021)** | Effect of biological factors on latency of pattern-reversal visual evoked potentials associated with cathode ray tubes and liquid crystal display monitors in normal young subjects | Healthy young adults, 21–29 years; n=30; Japan | Pattern reversal checkerboard; check size 30′; Michelson contrast ~95%; mean luminance ~50 cd/m²; monocular stimulation; reversal rate 1 Hz (2 rps); CRT and LCD displays | Oz–Fz derivation (10–20); ground Cz; band-pass 1–200 Hz; sweep 250 ms; 200 sweeps averaged; ≥2 reproducible recordings | P100 latency ↔ with biological factors; P100 latency ↑ with LCD vs CRT | P100 latency was not significantly affected by biological factors in young adults, but was longer when recorded using LCD compared with CRT displays |
| **Baumgarten et al. (2022)** | Fullfield and extrafoveal visual evoked potentials in healthy eyes: reference data for a curved OLED display | Healthy subjects ≥10 years; n=162; Germany | Pattern reversal checkerboard (full-field); check sizes 1.4° and 20.4′; Michelson contrast ~99%; mean luminance ~53 cd/m²; monocular stimulation; reversal rate 1.5 Hz; curved OLED display | Oz–Fz derivation (10–20); ground Cz; band-pass 1–100 Hz; sweep 250 ms; ≥100 sweeps averaged; impedance <5 kΩ | P100 latency ↓ until early adulthood then ↑ with age; P100 amplitude ↓ with age; P100 latency ↓ and amplitude ↑ in females | Age-related reference limits for full-field pattern-reversal VEP established using an OLED display; P100 latency shows U-shaped age dependency and amplitudes decrease with age, with consistently shorter latencies and larger amplitudes in females |
| **Kordek et al. (2022)** | Effect of Dioptric Blur on Pattern-Reversal and Motion-Onset VEPs as Used in Clinical Research | Healthy adults, 23–52 years; n=12; Germany | Pattern reversal checkerboard; check sizes 15′ and 60′; high-contrast black–white stimulus; mean luminance ~17 cd/m²; monocular stimulation; reversal rate 1 Hz (2 rps); induced dioptric blur | Oz derivation (10–20); reference A2; band-pass 0.1–100 Hz; ≥2 reproducible recordings | P100 latency ↑ with increasing dioptric blur; P100 amplitude ↓ with increasing dioptric blur | P100 latency increased and P100 amplitude decreased progressively with increasing levels of dioptric blur |
| **Thompson et al. (2023)** | ISCEV standard pattern reversal VEP development: paediatric reference limits from 649 healthy subjects | Healthy infants and children, 2 weeks–16 years; n=649 (large checks), n=403 (small checks); three European centres | Pattern reversal checkerboard; large checks ~50–60′, small checks ~12–15′; Michelson contrast ~95–98%; mean luminance ~54–80 cd/m²; CRT displays; binocular stimulation; reversal rates 1–3.75 s⁻¹ (centre- and age-dependent) | Oz–Fz derivation (10–20); sampling ≥0.96–1 kHz; bandwidth approx. 0.3–300 Hz; sweep 285–1066 ms; ≥2 replications or statistical significance testing | P100 latency ↓ with age until ~27 wks (large) / ~34 wks (small), then ↔; P100 amplitude ↔ with age (high variability) | Pooled paediatric ISCEV-standard prVEP reference limits established; sex differences not clinically meaningful; peak time matures rapidly in infancy and stabilises, amplitude highly variable |
| **Slapø et al. (2023)** | Relationship between function and structure in the visual cortex in healthy individuals and in patients with severe mental disorders | Healthy individuals and patients with severe mental disorders; healthy controls n≈307; adults; Norway | Pattern reversal checkerboard; high-contrast black–white stimulus; binocular stimulation; intervention-block pattern-reversal paradigm | Electrodes placed over occipital cortex (Oz region) with frontal reference; standard scalp EEG (10–20); band-pass compatible with clinical VEP; sufficient averaging to identify P100 | P100 amplitude ↑ with greater V1 cortical surface area; ↔ with age | P100 amplitude was positively associated with visual cortex surface area, while no consistent age-related effects on P100 parameters were observed |
| **Mirzaee Saba et al. (2023)** | P100 Wave Latency and Amplitude in Visual Evoked Potential Records in Different Visual Quadrants of Normal Individuals | Healthy adults, 18–35 years; n=40 (80 eyes); Iran | Pattern reversal checkerboard; check size 30′; Michelson contrast 85%; mesopic conditions; monocular stimulation; quadrant-specific visual field stimulation | Oz–Fz derivation (10–20); ground Cz; band-pass 1–100 Hz; ≥100 sweeps averaged; reproducible recordings | P100 latency ↑ in peripheral vs central quadrants; P100 amplitude ↓ in peripheral quadrants | P100 latency was longer and amplitude lower for peripheral visual field stimulation compared with central stimulation, with quadrant-dependent differences in P100 parameters |
| **Chen et al. (2023)** | A feasibility study for objective evaluation of visual acuity based on pattern-reversal visual evoked potentials and other related visual parameters with machine learning algorithm | Healthy young adults, 20–35 years; n=24 (48 eyes); China | Pattern reversal checkerboard; check sizes 5.7°, 2.6°, 1°, 34′, 15′, 7′; Michelson contrast 96%; mean luminance ~120 cd/m²; monocular stimulation; viewing distance 1 m; reversal rate 2 rps | Oz–Fz derivation (10–20); ground at earlobe; band-pass 1–100 Hz; sweep 250 ms; ~100 sweeps averaged; ≥2 reproducible recordings | P100 latency ↑ with decreasing visual acuity; P100 amplitude ↓ with decreasing visual acuity | P100 peak time lengthened and P100 amplitude decreased as visual acuity declined, with the strongest associations observed at 1° check size |
| **Utrobičić et al. (2023)** | Is the prolongation latency of visual evoked potentials a pathological sign in children with Down’s syndrome without ocular abnormalities? | Children with Down’s syndrome and age-matched healthy controls; 6–12 years; n=36 per group; Croatia | Pattern-reversal checkerboard; check size 1°; mean luminance not specified; viewing distance 50 cm; monocular stimulation; reversal rate 2 rps; refractive correction during testing | Oz–Fz derivation (10–20); ground earlobe; Tomey EP-1000; band-pass not specified; ~64 sweeps averaged; ≥2–3 repetitions | P100 latency ↑ in Down’s syndrome vs controls; P100 amplitude ↔ between groups | P100 latencies were significantly prolonged in children with Down’s syndrome despite absence of ocular abnormalities, while P100 amplitudes were comparable to healthy controls |

Supplementary Table 1. Characteristics of studies included in the scoping review

This table summarises studies included in the scoping review that investigated pattern-reversal visual evoked potentials (PR-VEPs) in relation to age and other biological or optical factors. For each study, details on the study population, stimulus and recording parameters, direction of effects on P100 latency and/or amplitude, and the main conclusions reported by the authors are provided. Only studies meeting the predefined inclusion criteria and methodological scope of the review are presented.
