# Supplementary material for: Physiological determinants of cortical P100 responses in pattern visual evoked potentials: a scoping review
Source: Front Neurosci. 2026 May 26;20:1821657. doi: 10.3389/fnins.2026.1821657 (PMC13246599; doi:10.3389/fnins.2026.1821657)
Supplement: Supplementary file 2 [file Table_2.docx]

| **Author (Year)** | **Title** | **Population** | **Stimulus parameters** | **Recording parameters** | **Direction of effects** | **Main conclusions (authors)** | **ISCEV compliance / Notes** |
| --- | --- | --- | --- | --- | --- | --- | --- |
| **Larsen (1979)** | Axial length of the emmetropic eye and its relation to head size | Healthy adults; emmetropic eyes; Denmark (abstract-only) | No VEP (ultrasonographic axial length measurement) | Not applicable | Not applicable | Axial eye length was significantly correlated with head circumference, head length, head breadth, and body height | Non-VEP study; outside VEP-based review scope |
| **de Graaf et al. (1985)** | Standards for the assessment of visual evoked potentials in an ethnically heterogeneous adult population | Healthy adults, 15–73 years; n=276; South Africa; ethnically heterogeneous | Pattern-reversal checkerboard; check size ~25–28′; contrast 100%; luminance not reported in ISCEV 2016 format; monocular; reversal rate 1 Hz | Occipital scalp recording with frontal reference Fz (10–20); band-pass ~1.6–32 Hz; sweep 300 ms; 128 sweeps | P100 latency ↓ until mid-adulthood then ↑ with age; P100 latency ↓ in black vs white subjects; P100 amplitude ↑ in females | P100 latency showed a non-linear relationship with age and differed between ethnic groups; P100 amplitude higher in females | Published long before ISCEV 2016; outside predefined 2016–2025 scope |
| **Guthkelch et al. (1987)** | The relationship of the latency of the visual P100 wave to gender and head size | Healthy adults; 8 males and 8 females; country not specified (abstract-only) | Pattern-reversal VEP; stimulus parameters not reported (abstract-only) | Recording parameters not reported (abstract-only) | P100 latency ↑ with larger head circumference; ↔ with gender | P100 latency correlated more strongly with head circumference than with gender; head length and N70 latency showed no significant associations | Abstract-only; insufficient methods for ISCEV assessment; published long before ISCEV 2016; outside predefined 2016–2025 scope |
| **Gregori et al. (2006)** | VEP latency: Sex and head size | Healthy adults; Italy | Pattern-reversal checkerboard; stimulus parameters reported per pre-2016 practice | Occipital recording with frontal reference (10–20); recording/averaging per pre-2016 practice | P100 latency ↑ with larger head size; P100 latency ↑ in males vs females | P100 latency longer with larger head size and in males vs females | Published long before ISCEV 2016; outside predefined 2016–2025 scope |
| **Solanki et al. (2013)** | Visual evoked potential: Head size, sex, and BMI | Healthy medical students, 16–18 years; n=48 (24 males, 24 females); India | Pattern-reversal checkerboard; stimulus size reported as 8″ × 8″; contrast/luminance not reported in ISCEV 2016 format; monocular; reversal rate ~1.7 Hz | Occipital recording with frontal reference (10–20); RMS EMG EP MARK II; recording/averaging per pre-2016 practice | P100 latency ↑ with larger head circumference; ↔ with sex and BMI | P100 latency was associated with head circumference; sex and BMI showed no significant effects | Pre-ISCEV 2016 reporting; outside predefined 2016–2025 scope |
| **Kothari et al. (2013)** | Association of Height With Pattern Reversal Visual Evoked Potentials | Healthy adults, 40–79 years; n=200 (400 eyes); Central India | Pattern-reversal checkerboard; full-field; check size ~2.2°; field ~18°; contrast 80%; mean luminance ~59 cd/m²; monocular; reversal rate 1 Hz | Oz active; Fz reference; Cz ground (10–20); band-pass 2–100 Hz; sweep 300 ms; 200 sweeps; ≥2 trials | P100 latency ↑ with height; P100 amplitude ↓ with height | P100 latency increased and P100 amplitude decreased with increasing height | Published prior to ISCEV 2016; outside predefined 2016–2025 scope |
| **Dave et al. (2014/2017)** | Analysis of gender based differences in pattern reversal visual evoked potentials among healthy subjects of North India | Healthy adults, 18–25 years; n=100 (50 males, 50 females); India | Pattern-reversal checkerboard; check size 15′; contrast 70%; mean luminance 50 cd/m²; reversal rate ~2 rps; monocular stimulation | Oz–Fz (10–20); band-pass 1–100 Hz; 150 sweeps averaged | P100 latency ↑ in males vs females; P100 amplitude ↓ in males vs females | Females show shorter P100 latencies and higher P100 amplitudes compared with males | ISCEV reference limited to 2009 update; study conducted/reported before ISCEV 2016 standard |
| **Akın et al. (2015)** | Visual and brainstem auditory evoked potentials in children with obesity | Children with obesity and age-matched healthy controls; Turkey | Pattern-reversal checkerboard; monocular; reversal every 20 ms; stimulus parameters not reported in ISCEV-recommended format | Occipital scalp recording with frontal reference (10–20); recording/averaging not reported in ISCEV-recommended format | P100 latency ↑ in children with obesity vs controls; P100 amplitude ↔ between groups | P100 latencies prolonged in children with obesity vs controls; P100 amplitudes comparable | Pre-ISCEV 2016 reporting; no declaration/implementation of ISCEV 2016; outside predefined 2016–2025 scope |
| **Sharma et al. (2015)** | Visual Evoked Potentials: Normative Values and Gender Differences | Healthy young adults, 17–20 years; n=100; India | Pattern-reversal checkerboard; high-contrast black–white; viewing distance 100 cm; monocular; reversal rate ~2 rps | Oz–Fz (10–20); ground Cz; band-pass 1–100 Hz; sweep 250 ms; ≥100 sweeps; ≥2 reproducible recordings | P100 latency ↔ with sex; P100 amplitude ↑ in females | P100 latency did not differ between sexes; P100 amplitude higher in females | Based on ISCEV 2009 update; conducted/published before ISCEV 2016; outside predefined 2016–2025 scope |
| **Kovarski et al. (2016)** | Brief Report: Early VEPs to Pattern-Reversal in Adolescents and Adults with Autism | Adolescents and adults with ASD and typically developing controls; ~12–40 years; small cohorts | Pattern-reversal checkerboard; large checks (~1.9° × 1.5°); high-contrast; binocular; reversal rate 1 Hz | 64-channel EEG (BioSemi); Oz/O1/O2/POz/PO3/PO4; band-pass 0.5–30 Hz | P100 amplitude ↓ with age; P100 amplitude ↓ in ASD vs controls; P100 latency ↔ with age and diagnosis | P100 amplitude reduced with increasing age and lower in ASD vs controls; P100 latency not significantly different between groups | Clinical group comparison; not designed for ISCEV normative reference despite pattern-reversal VEP use |
| **Lisicki et al. (2016)** | Familial history of migraine influences habituation of visual evoked potentials | Migraine without aura patients and healthy volunteers with and without first-degree family history of migraine; adults; n=60; Argentina and Belgium | Pattern-reversal checkerboard; check size 14′; continuous stimulation; reversal rate 3.1 Hz; monocular; viewing distance 1 m | Oz active; Fz reference; ground forearm (10–20); band-pass 1–100 Hz; sweep 200 ms; six consecutive blocks of 100 responses | P100 latency ↔ between groups; N1–P1 amplitude ↓ and habituation ↓ in migraine and relatives | Lack of VEP habituation and reduced first-block amplitudes were observed in migraine patients and relatives | Non-ISCEV habituation paradigm; no declaration/implementation of ISCEV 2016+; outside predefined 2016–2025 scope |
| **Gupta et al. (2017)** | Visual and brainstem auditory evoked potentials in obese and overweight individuals | Overweight/obese adults and healthy controls; India | Pattern-reversal checkerboard; check size ~54.6′; mean luminance ~50 cd/m²; monocular; reversal rate 2 Hz | Occipital recording with frontal reference (10–20); band-pass 2–100 Hz; averaging per pre-2016 practice | P100 latency ↑ in obese/overweight vs controls; P100 amplitude ↔ between groups | P100 latencies prolonged in obese/overweight vs controls; P100 amplitudes not significantly different | No ISCEV 2016 declaration; follows pre-2016 conventions; outside predefined 2016–2025 scope |
| **Torres-Espínola et al. (2018)** | Visual evoked potentials in offspring born to mothers with overweight, obesity and gestational diabetes | Infants; PREOBE cohort; cVEP at 3 and 18 months; Spain | Pattern-reversal checkerboard; contrast 100%; binocular; spatial frequencies 2°, 1°, 30′, 15′, 7.5′; mean luminance ~39 cd/m²; reversal rate ~2.1 s⁻¹ | O1/Oz/O2 (active), Fz (reference), Cz (ground); band-pass 1.5–100 Hz; CRT display; awake infants | P100 latency ↑ in offspring of gestational diabetes mothers at 18 months (especially smaller checks); P100 amplitude ↔ between groups | At 18 months, offspring of gestational diabetes mothers showed prolonged P100 latencies vs controls; P100 amplitudes did not differ | Non-ISCEV developmental cVEP protocol; ISCEV reference limited to 1995 standard |
| **Singh (2018)** | Acute Effect of Electromagnetic Waves Emitted from Mobile Phone on Visual Evoked Potential in Adult Male: A Preliminary Study | Healthy adult males, 20–40 years; n=9; India | Pattern-reversal checkerboard; check size ~32′; contrast ~67%; monocular; reversal rate 1 Hz; viewing distance 1 m; mobile phone exposure paradigm | RMS EMG EP MK-2; Cz active with references O1–O2; ground Fz; 100 sweeps averaged | P100 latency ↑ after mobile phone exposure; P100 amplitude ↔ | P100 latency increased after short-term exposure; amplitude changes not statistically significant | Non-ISCEV montage/protocol; no declaration/implementation of ISCEV 2016; outside predefined 2016–2025 scope |
| **Dziadkowiak & Podemski (2019)** | Impact of Hyperventilation and Sleep Deprivation upon Visual Evoked Potentials in Patients with Epilepsy | Patients with newly diagnosed epilepsy and healthy controls; adults; Poland; n=81 patients, n=42 controls | Pattern-reversal checkerboard (check size ~1.1°) and uniform flash stimulation; full-field; binocular; flash frequencies 1.88 Hz and 15 Hz; mixed transient/steady-state paradigms | Occipital midline electrode above external occipital protuberance with frontal reference (10–20); Nicolet CA-1000; band-pass not specified; repeated recordings | P100 latency ↔ with checkerboard stimulation; P100 latency ↑ with flash stimulation after hyperventilation/sleep deprivation | Checkerboard VEP parameters did not differ between patients and controls; flash VEP latency changes observed after activation procedures | Non-ISCEV mixed checkerboard/flash paradigm; no declaration/implementation of ISCEV 2016+; outside predefined 2016–2025 scope |
| **Mahjooba et al. (2019)** | Normative values of visual evoked potentials in Northeastern Iran | Healthy adults, 18–30 years; n=59; Iran | Pattern-reversal VEP; check sizes 15′ and 60′ | Oz–Fpz (10–20); ground Cz | P100 latency ↑ with smaller check size; ↔ with sex and interocular comparison | P100 latency depends on check size; no significant sex- or interocular-related differences in P100 latency; N75–P100 amplitude higher in binocular recordings and in females | ISCEV reference limited to 2009 update; no explicit reference to ISCEV 2016/2021/2025 |
| **Jensen et al. (2019)** | Neural correlates of early adversity among Bangladeshi infants | Infants (6 months; n=91) and children (36 months; n=112); Bangladesh (Dhaka) | Pattern-reversing checkerboards; binocular; fixation-contingent presentation monitored by eye tracking | 128-channel EEG; sampled 500 Hz; band-pass 0.3–30 Hz; epochs −100 to 300 ms; electrode 75 (Oz) extracted; referenced to Cz at acquisition and re-referenced to average reference | P100 amplitude ↑ with concurrent/prospective cognitive outcomes; P100 latency ↔ with cognitive outcomes | P1 amplitude positively associated with cognitive outcomes; no consistent associations for P1 latency | Non-ISCEV ERP/cVEP with high-density EEG and average reference; ISCEV 2016 used only to define P1 window |
| **Nieto-Ruiz et al. (2019)** | Cortical Visual Evoked Potentials and Growth in Infants Fed with Bioactive Compounds-Enriched Infant Formula | Infants assessed at 3 and 12 months; RCT (COGNIS); Spain | Pattern-reversal checkerboard; contrast 100%; binocular; spatial frequencies 2°, 1°, 30′, 15′, 7.5′; mean luminance ~39 cd/m²; reversal rate ~2.1 s⁻¹ | O1/Oz/O2 (active), Fz (reference), Cz (ground); band-pass 1.5–100 Hz; CRT display | P100 latency ↓ with age; P100 latency ↑ and P100 amplitude ↓ in formula-fed vs breastfed infants | Breastfed infants had shorter P100 latencies and higher P100 amplitudes vs formula-fed infants at 3 and 12 months | Non-ISCEV developmental cVEP protocol; no explicit reference to ISCEV 2016/2021/2025 |
| **Narra et al. (2020)** | Assessment of visual and auditory evoked potentials in young obese males | Young obese males and healthy controls; India | Pattern-reversal checkerboard; contrast ~70%; monocular; reversal rate ~1.71 Hz; stimulus parameters not in ISCEV 2016 format | Occipital recording with frontal reference (10–20); band-pass 2–100 Hz; averaging per pre-2016 practice | P100 latency ↑ in obese males vs controls; P100 amplitude ↔ between groups | P100 latencies prolonged in obese males vs controls; P100 amplitudes did not differ | No ISCEV 2016 declaration; follows pre-2016 conventions; outside predefined 2016–2025 scope |

Supplementary Table 2. Characteristics of studies excluded from the scoping review

This table lists studies identified during the literature search that were excluded from the scoping review. Reasons for exclusion include publication outside the predefined time frame, non-ISCEV or pre-ISCEV methodological standards, insufficient reporting of stimulus or recording parameters, use of non-pattern-reversal VEP paradigms, abstract-only publications, or study designs not aligned with the objectives of the review. Key characteristics and notes explaining exclusion are provided for transparency.
